# Basis of preventive and non-pharmacological interventions in asthma

**DOI:** 10.3389/fpubh.2023.1172391

**Published:** 2023-10-18

**Authors:** Vicente Javier Clemente-Suárez, Juan Mielgo-Ayuso, Domingo Jesús Ramos-Campo, Ana Isabel Beltran-Velasco, Ismael Martínez-Guardado, Eduardo Navarro Jimenez, Laura Redondo-Flórez, Rodrigo Yáñez-Sepúlveda, Jose Francisco Tornero-Aguilera

**Affiliations:** ^1^Faculty of Sports Sciences, Universidad Europea de Madrid, Madrid, Spain; ^2^Studies Centre in Applied Combat (CESCA), Toledo, Spain; ^3^Department of Health Sciences, Faculty of Health Sciences, University of Burgos, Burgos, Spain; ^4^LFE Research Group, Department of Health and Human Performance, Faculty of Physical Activity and Sport Science-INEF, Universidad Politécnica de Madrid, Madrid, Spain; ^5^Psychology Department, Universidad Antonio de Nebrija, Madrid, Spain; ^6^BRABE Group, Department of Psychology, Faculty of Life and Natural Sciences, Universidad Camilo José Cela, Madrid, Spain; ^7^Universidad Simón Bolívar, Facultad de Ciencias de la Salud, Barranquilla, Colombia; ^8^Department of Health Sciences, Faculty of Biomedical and Health Sciences, Universidad Europea de Madrid, Madrid, Spain; ^9^Faculty of Education and Social Sciences, Universidad Andres Bello, Viña del Mar, Chile

**Keywords:** asthma, environment, microbiota, nutrition, physical exercise, ergo nutritional, psychology

## Abstract

Asthma is one of the most common atopic disorders in all stages of life. Its etiology is likely due to a complex interaction between genetic, environmental, and lifestyle factors. Due to this, different non-pharmacological interventions can be implemented to reduce or alleviate the symptoms caused by this disease. Thus, the present narrative review aimed to analyze the preventive and non-pharmacological interventions such as physical exercise, physiotherapy, nutritional, ergonutritional, and psychological strategies in asthma treatment. To reach these aims, an extensive narrative review was conducted. The databases used were MedLine (PubMed), Cochrane (Wiley), Embase, PsychINFO, and CinAhl. Asthma is an immune-mediated inflammatory condition characterized by increased responsiveness to bronchoconstrictor stimuli. Different factors have been shown to play an important role in the pathogenesis of asthma, however, the treatments used to reduce its incidence are more controversial. Physical activity is focused on the benefits that aerobic training can provide, while physiotherapy interventions recommend breathing exercises to improve the quality of life of patients. Nutritional interventions are targeted on implement diets that prioritize the consumption of fruits and vegetables and supplementation with antioxidants. Psychological interventions have been proposed as an essential non-pharmacological tool to reduce the emotional problems associated with asthma.

## 1. Background

**Figure 1:**
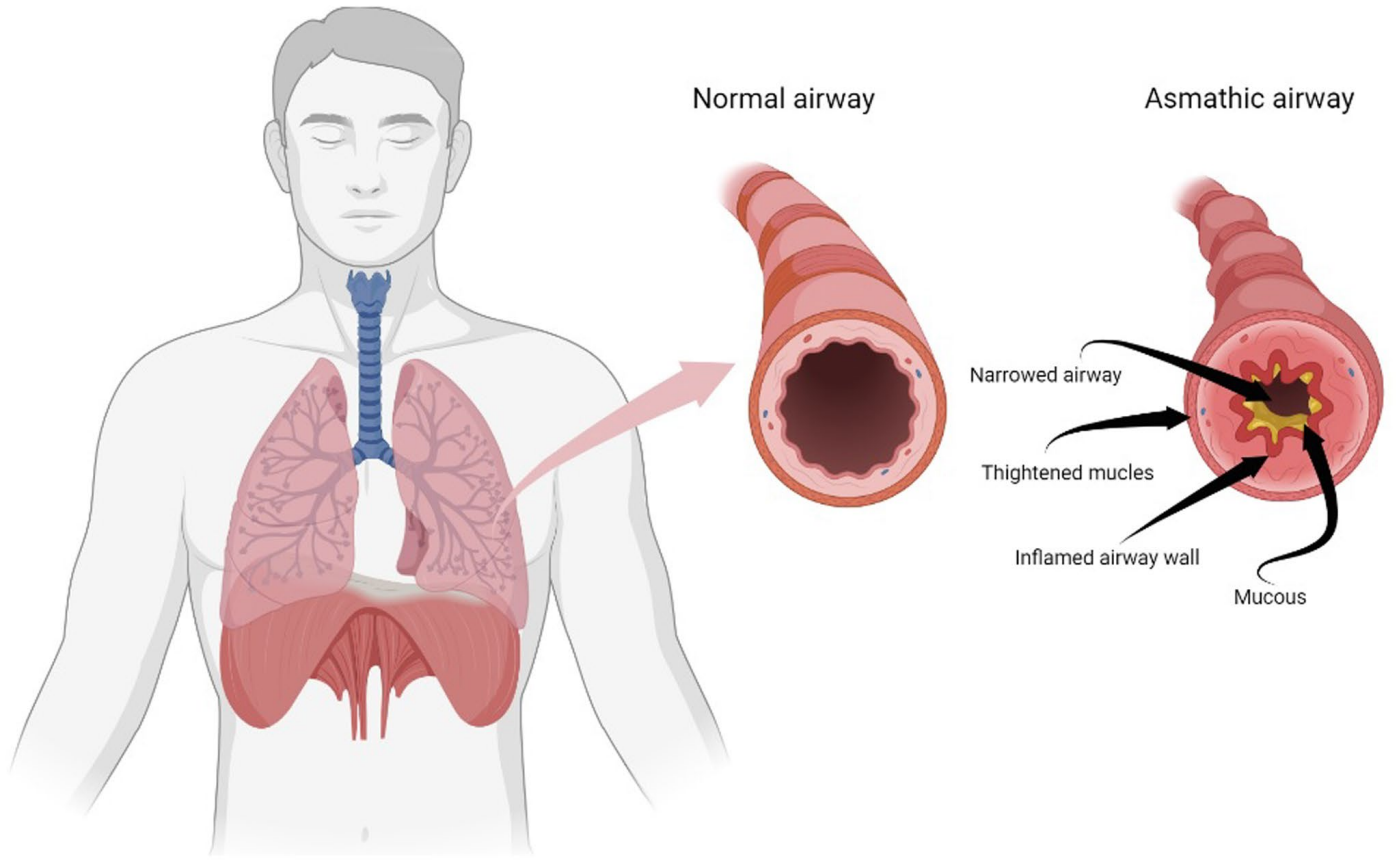
Airway changes in asthma. Made with Biorender Software.

Asthma affects children and adults, being a chronic disease. The passageways that carry air to the lungs narrow due to inflammation and compression of the muscles that surround the small airways (Figure 1). This causes the symptoms of asthma: cough, wheezing, shortness of breath, and chest tightness. These symptoms are intermittent and are often aggravated at night or with exercise. Triggers vary from person to person but include viral infections (colds), dust, smoke, fumes, weather changes, grass and tree pollens, animal fur and feathers, synthetic soaps made with compounds such as glycols, parabens or formalin, and perfumes (1).

Asthma is one of the main non-communicable diseases (NCDs), affecting children and adults. It is estimated that in 2016 there were more than 339 million people with asthma worldwide and that in 2019, asthma affected 262 million people and caused 461,000 deaths (2). In the review of Eagan et al., the estimation of adult asthma is 4.6 per 1,000 person-years in women and 3.6 per 1,000 person-years in men. In cohort studies, the estimations among general population was higher, respectively 5.9 and 4.4 per 1,000 person-years in women and men, with an adult incidence slightly higher in women than men, presenting a trend toward a higher incidence with age (3). In addition, the European Respiratory Health Survey (ECRHS) found that in a population aged 30–54 years of Sweden, Norway, Denmark, Iceland, and Estonia the incidence rate of asthma ranged 1.5–2.2·1,000 person-yrs − 1, with a higher incidence range among females (4).

Asthma has been associated with very different risk factors. The chance of developing asthma is higher if other family members are also asthmatic, particularly close relatives such as parents or siblings, and it is also more common in people with other allergies. Certain events that occur early in life can affect the developing lungs and increase the risk of asthma. Among these factors are low birth weight, prematurity, exposure to tobacco smoke and other sources of air pollution, and viral respiratory infections. In addition, it is believed that exposure to several allergens and irritants in the environment may increase the risk of asthma, as indoor air pollution and pollen, house dust mites, molds, and exposure to work to chemicals, fumes, or dust. Along these lines, urbanization has been associated with an increase in the prevalence of asthma, probably due to several factors related to lifestyle, as well as being overweight, which has been associated with a greater risk of suffering from asthma (5).

Asthma is considered to have no cure, proposing a treatment with inhaled medications can help control the disease, as well as make it easier for asthmatic people to lead a normal and active life (6). however, different non-pharmacological therapeutic approaches can improve the symptoms of asthmatic patients, and that in many cases are not applied. For this reason, this narrative review was conducted to analyze the basis of non-pharmacological interventions in asthma.

To reach the study purpose, a consensus and critical review were conducted, analyzing primary sources such as academic research and secondary sources such as databases, web pages, and bibliographic indexes, following procedures of previous critical narrative reviews (7–10). We reviewed MedLine (Pubmed), Cochrane (Wiley), Embase, and CinAhl. Databases the MeSH-compliant keywords of asthma and intervention, nature, early exposure, environment, pathogenesis, GUT, microbiota, stress, metabolic health, physical activity, physiotherapy, nutrition, ergogenic, and psychology. We used manuscripts published from 1 January 2012 to 10 May 2022, although previous studies were included to explain some information in several points of the review. We used the following exclusion criteria in line with previous reviews (11, 12): i) research outside the period analyzed, ii) presented topics out of the review scope, iii) Unpublished studies, books, conference proceedings, abstracts, and Ph.D. dissertations. We used all the studies that met the scientific methodological standards and had implications with any of the subsections of the present review. The treatment of the information was performed by all the authors of the review, and finally, the articles selected were discussed to write the present review.

## 2. Exposure to environmental, climatological and natural conditions

The prevalence of allergen-related diseases is increasing every year and is already considered a public health problem by many professionals in this area (13). It is estimated that between 30 and 40% of the world’s population is affected by an allergy and frequently suffers from allergic rhinitis symptoms (14). This condition usually appears with exposure to environmental allergens such as pollen, facilitating the presence of sneezing, rhinorrhea, tearing, itching, and mild to severe nasal obstruction (15).

Recent studies have been able to demonstrate that the pollen that is transported through the air is increasing (16). The increase in pollen production in plants that are pollinated by the wind has made it easier for the air we breathe to have a higher concentration of pollen (17). This could be explained by the fact that plants are producing more pollen when temperatures are high, which makes it easier for this to happen not only in natural environments but also in urban environments (18, 19).

### 2.1. Allergen tolerance

An important factor that may be affecting the production of potentially allergenic substances in plants is climate change. This is due to climate change affecting all plant processes, including the flowering process, and pollen production (20). This is because plants are subjected to high amounts of carbon dioxide (CO2) (21). This causes plants to produce proteins that are intended to protect the planet from climate change (22). f these natural processes are compared in their production in the ‘90s, we can see that nowadays they are more than 20 days earlier and last at least 8 to 10 days (23). On the other hand, the derived allergic symptoms are more intense and severe since plants and trees have increased their pollen production by more than 30% compared to previous decades. Moreover, the forecast is that this will continue to increase over the next 40–50 years (24). All this is important because a person’s immune system tends to become less reactive as age increases, which means that the symptomatology associated with allergens is usually reduced (25). However, climate change is causing this natural, innate protection in people to disappear. Not only will seasonal allergic symptoms remain, but they will worsen and last longer (26). Proof of this is that, at present, almost 20% of the adult population between the ages of 50 and 65 are suffering from allergies for the first time in their lives (27).

Examples of the impact of climate change are the results of studies in different countries. In the Netherlands, a direct relationship has been verified between pollen levels and the advancement of high temperatures that cause the pollen season to extend its duration and its levels of pollen concentration in the air (28). In addition, evidence of pollen transport over long distances has been recorded (29). Thus, it is known that pollen has been transported from Morocco, the Iberian Peninsula (Spain), and the Sahel to the Canary Islands. In other words, pollen travels up to more than 2,000 kilometers and is responsible for respiratory symptoms (30). In Beijing, we also find large amounts of haze that hinder the free circulation of the wind in the middle troposphere and therefore favor the accumulation of allergens in the air. The same applies to pollution. The progressive increase that has occurred in recent years in this issue also causes the number of allergies to increase, as it directly affects the presence of more allergens in the air (31).

Actually, the food allergies account for a prevalence of up to 45% in Europe, according to data collected by EAACI (European Academy of Allergy and Clinical Immunology). Worldwide, it is estimated that between 10 and 40% of the population suffers from this pathology, according to data collected by WAO (World Allergy Organization). More than 20% of these diagnoses are found in developed countries, although the type of food and other variables are modulators in the expression of allergy (32, 33). Furthermore, it is well known that food allergies have increased markedly in recent decades, ranging from 1 to 3% in adults and from 4 to 6% in children (32). These allergies appear during the first years of life and have a great impact on the life of children and families because of the implications in all areas of the child (family, school, social) and because of the high number of adverse reactions that appear at the time of ingestion of food whose allergy is already known, and at the time of ingestion of new food that can cause unknown and highly severe symptoms, such as anaphylaxis (33).

The origin of this type of allergy is a complex issue. Our body detects a protein-mediated by immunoglobulin E (IgE), which is present in the blood, and which is concentrated in greater quantities when there are allergic reactions (34). When this protein is detected, our body secretes IgE to fight and attack it. This release into the bloodstream causes histamine to be released, which favors the appearance of allergic symptoms. However, it is not easy to know why this process occurs (35). There may be a genetic factor when there is a family history of allergy, but it could also be explained by the way food is cooked or processed, which can facilitate the likelihood of food allergies (36).

In children, a very high percentage, 90%, of allergies come from cow’s milk. Other foods found in this group are eggs, nuts, peanuts, fish, wheat, and soya (37). However, any food can cause allergies in children, and this will only be known during the first intake of the food (38).

At present we do not know exactly what are the processes that trigger the allergic response, but we can state that it is a relationship between genetic factors and environmental factors (39). We know that it is not possible to act with generic factors. However, it is possible to prevent some environmental factors (31). In this sense, exposure to allergens from the first moments of life is an essential factor so that our immune system does not detect them as a foreign body to be fought against (40).

### 2.2. Early life exposures

In this line, in the last decades, a method of upbringing is being used in which children are deprived of exposure to the natural allergens with which human beings have historically coexisted (41). Knowing that the genetics of human beings has not changed in the last 100 years, lifestyle and home conditions such as excessive protection and even hygiene, favor that the child is not exposed to certain allergens from an early age and therefore, sensitization to pollens, mites and food appears favoring the emergence of allergies. The same is true when the child is not exposed from an early age to allergens that are present in nature such as pollen, mold or animal hair (42). This will determine the presence of allergies and intrinsic asthma, associated with the initial allergy (43). In this regard, it is relevant to note that the body is naturally prepared to fight against potentially dangerous substances. The immune system is composed of antibodies, white blood cells, complement proteins, mast cells and other substances that enable defense against adverse elements called allergens. It is at the first exposure to these elements that the immune system produces an antibody called immunoglobulin E, which causes sensitivity to the allergen and triggers a chain reaction. When this exposure is naturally delayed or inhibited from the earliest years of life, the chances of more aggressive allergies are greatly increased (44–46).

The immune system must be prepared to provide an adequate response to antigenic stimuli that occur during vital development (47). However, the recent overprotection of newborns makes the immune system more vulnerable because it is not subjected to adequate concentration levels of various pathogens and there is no stimulation to activate the proper functioning of the immune system (48). Excessive hygiene and the absence of germs together with early vaccinations and other measures to avoid common infections are actions that are directly related to the child’s immune system not being activated, facilitating the appearance of allergies (49, 50). All this, together with the increasing levels of pollution and the lack of rain that we are suffering, is causing allergies to increase significantly and asthma in children is exacerbated (51).

The WHO (World Health Organization) indicates that more than 90% of the child population is subjected to very high levels of pollution, above what is legally permitted, which also favors the increase of asthma in children (52). This is important considering that in children under 5 years of age, bronchial hyperreactivity that triggers asthma is the chronic disease that causes the highest number of admissions to the hospital network (53). In this line, recent studies indicate that infant-juvenile patients are those who suffer the most severe symptoms (54). In Latin America, the prevalence of this disease in children is over 17%, reaching up to 30% in some populations (55). In Europe, the countries with the highest prevalence of asthma and childhood allergies are Germany, Ireland, the United Kingdom, and Finland, reaching 25% of the affected child population. Along the same lines, another interesting fact is that in 2019 there was an estimated affectation of more than 260 million people with asthma worldwide and more than 460,000 deaths directly or derived from respiratory complications (56).

## 3. Close environment and asthma pathogenesis

Recent researchers have proposed that environmental exposures can significantly influence the phenotype of allergic diseases, including asthma (45). In this line, the prevalence of this disease is increasing, mainly due to genetic changes related to the inflammatory pathways (57). However, The International Study of Asthma and Allergies in Childhood established that other factors such as the socio-economic conditions of the different regions or countries can generate different environmental conditions that are precursors to asthma, such as air pollution, dietary patterns, or viral infections (58). In this line, early childhood is considered a particularly vulnerable period as the immune system is still developing (59), showing how the increase in allergic diseases is due to the loss of symbiotic relationships with parasites and bacteria that allow the immune system to remain alert (60). Thus, some factors such as the low interaction of children with the environment may produce a reduce environmental microbial exposures, and the use of a greater amount of antibiotics, among others, favor the development of respiratory diseases such as asthma (61).

In this line, when it was compared children who lived on farms with a control group who in the prevalence of asthma and atopy in function to the diversity of microbial exposure, it was shown how the exposition to a larger variety of environmental microorganisms correlated with lower prevalence asthma (61). Moreover, Feng et al. (62) reported similar results establishing a significantly lower prevalence of asthma in Chinese children living in rural areas compared to those living in an urban environment (2.8% vs. 29.4%, respectively). In the American population, it was observed that children living on smaller, traditional farms were exposed to a higher quantity and quality of microbial diversity than children living on more modern farms, showing a lower prevalence of asthma (5% vs. 23%, respectively) (63). In this fact, Riedler et al. (64), showed similar results to previous studies, indicating the importance of the time of exposure to microbial diversity during the first and fifth years of life to achieve a protective effect against asthma; and Lampi et al. (65) showed in their prospective birth cohort study in Finland population that a farming environment during infancy is associated with higher lung function (measured trough forced vital capacity and forced expiratory volume in 1 s) and lower asthma prevalence in adulthood. To support this, Genuneit et al. (66) showed in a meta-analysis that living under rich microbial conditions could reduce 25% asthma risk occur. Despite the results shown by the different investigations, the environmental microbiome may play a significant role in asthma pathogenesis, mechanisms behind this fact remain undefined (67). However, some authors have hypothesized that the differences in the skin microbiome and the nasal microbiota observed between both scenarios (rural and urban) could explain this pathological mechanism. In this line, Lehtimäki et al. (68) observed in the Copenhagen Prospective Studies on Asthma in Childhood that the levels of several cytokines and chemokines measured in nasal mucosal samples from subjects aged 1 month and plasma from subjects aged 6 months differed by rural or urban area. However, this fact was insufficient to conclude that asthma pathogenesis is explained by these results.

However, it is possible that exposure to the natural environment negatively affects the human microbiome and its immunomodulatory capacity, inducing the development of some type of allergy or asthma. In this line, Rufo et al. (69) carried out a longitudinal birth cohort study on Portuguese children, and their results showed that children who lived close to an environment with more green spaces at birth have a protective effect on the development of asthma. However, children who live close to a greater number of animals appear to be associated with a higher risk to develop this disease. Conversely, Dadvand et al. (70) conducted a cross-sectional study on Spanish children and reported that living close to parks was associated with a 60% higher relative prevalence of asthma. Concerning this, similar results in asthma prevalence associated with greener areas were found by Andrusaityte et al. (71) in Lithuanian children. In this regard, several investigations have been stated that protective effects of green environments could be associated with the autonomic nervous system, leading to stress reduction, lower heart rate and lower allostatic load (72, 73). However, further research is necessary to understand possible interactions and mechanisms between these environmental factors and the development of asthma in the early stages of life.

Furthermore, the indoor microbial environment (e.g., crowding, family size, daycare, and pet care) has been established as another determining factor in strengthening the immune system. In this line, several studies have shown that daycare attendance in early life was correlated with decreased asthma rates at school age (74) and adolescence (75). However, exposure to pets (e.g., dogs and cats) has been implicated as both a risk and a protective factor for developing asthma (76). In this line, in a cohort study conducted with Swedish children, it was reported how exposure to dogs and farm animals during the first year of life reduces the risk of asthma at age 6 years (77). Moreover, from a molecular view, it has been stated that endotoxins from the cell walls of gram-negative bacteria play a key role in protective effects for allergy and possibly asthma (78). Regarding this fact, higher endotoxin concentrations were significantly associated with having pets and more than 4 persons living in the same home according to the analysis of 3 European countries included in the AIRALLERG study (79). Thus, endotoxins could be essential to exert a protective function against the development of allergies and asthma. In this line, associations have been established between contact with pets during the first years of life and the increase in this parameter (45). In this line, for primary prevention of asthma and allergy, Lødrup Carlsen et al. (76) reported in their meta-analysis that there is not enough evidence to discourage parents from allowing children to have contact with pets in the first years of life, since it has been observed that it does not increase the risk of allergies or asthma.

## 4. Gut microbiome and asthma pathogenesis

The human body is made up of bacteria, viruses, protozoa, fungi, and archaea that live in balance, generating perfect body homeostasis. The above form the microbial tissues, which are found in the walls of tissues as important as the oral walls, nasopharynx, pulmonary, and gut. As so, the human body is inhabited by 10 times more microorganisms than the number of cells that our body counts, affecting many different life processes and guaranteeing homeostasis. Alterations in the microbiota and its composition can generate dysbiosis. This can be of multifactorial origin, but one of the most important is exposure to the environment. In this line, there is a strong impact on allergy and asthma regarding microbiome environmental exposure.

The first reports regarding the environmental microbiome as a protective component came from Riedler et al. (64). Authors determined that children living on Alpine farms, who had contact with farm animals and consumed unpasteurized milk, were less likely to have asthma, and allergic rhinitis, and were less atopic than their peers from non-farming families (64). In the upcoming years Braun-Fahrländer et al. (78), suggested that gram-negative bacterial endotoxins in samples of dust from the child’s mattress correlated inversely with the prevalence of atopy measured by allergen-specific IgE level and with bronchial asthma and cytokine production by peripheral blood leukocytes. In the same line, one of the most recent studies is that by Gozdz, et al. (80), studying the Hutterites and the Amish communities. Authors found between both populations that there was a huge difference in the prevalence of allergic diseases among children. In this line, the levels of bacterial endotoxin in the dust from the Amish houses are sevenfold that from the Hutterites’ houses, presenting a different bacteriological composition. Being the bacteriological composition of the dust. Authors exposed that the Amish’s dust exposure prevented the development of asthma in mice, but the dust from Hutterites’ houses induced bronchial hyperresponsiveness (80). Furthermore, a cross-sectional study in 2017 evaluated a children group of 6–12 years old living in rural areas and small towns of Europe. Authors suggested that in the rural environment the microbial diversity and the number of bacteria and fungi are significantly higher than in other areas (81). According to the authors “This specific microbial cocktail stimulates our immune system to protect against the development of allergies, thus, the more bacteria and fungi in the environment the less likely bronchial asthma occurs (82).” In urban environments in which exposure to microbial agents is lower than in rural ones, the authors analyzed exposure to domestic animals. They conclude that children who lived among home pets’ allergens in their first year of life are less likely to develop asthma, suggesting that bacteria present in the environment could play the role of adjuvant inducing immune tolerance to allergens (83).

The above studies strongly suggest that a microorganism-rich and natural environment offers protection against respiratory diseases such as asthma and allergies, with the ability to modify immune maturation in early life. This is mainly explained throw the activation of airway epithelial cells which are mediated by toll-like receptor 4 (TLR4) and nuclear factor κB (NF-κB) (84). Therefore, the secretion of pro-inflammatory mediators like chemokine CCL20 and granulocyte–macrophage–colony-stimulating factor is produced, necessary for the recruitment and maturation of dendritic cells. For asthma, the authors suggest that airway exposure to endotoxins inhibits the activation of NF-κB by the increase in the synthesis of its attenuator, enzyme A20, being this one therapeutic key target for asthma prevention (85).

**Figure 2:**
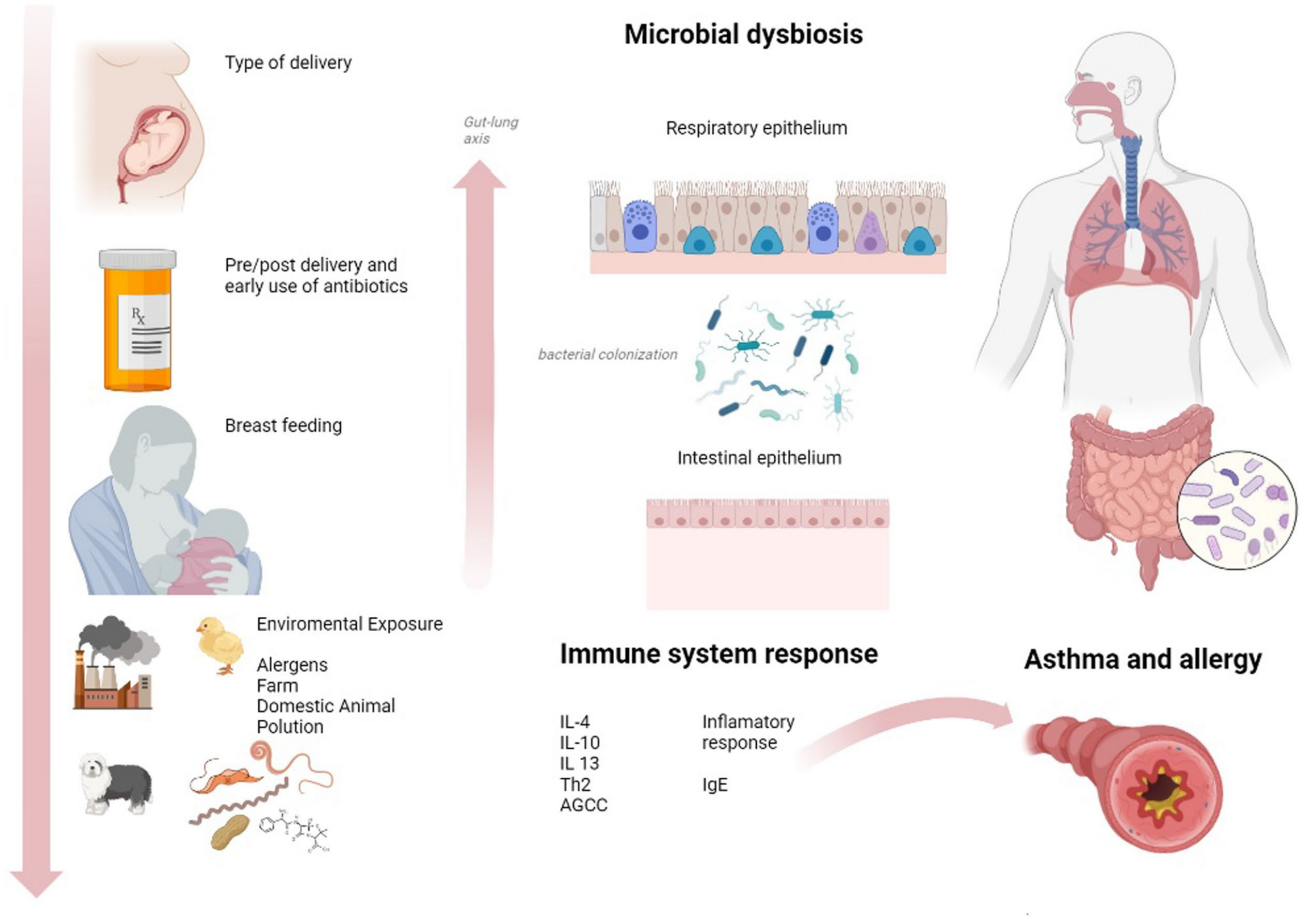
Contextual factors that affect the microbiota and asthma pathogenesis. Made with Biorender Software.

However, the interactions between external agents and the microbiota are more heterogeneous and complex than we think, and future research will expose the receptors, agents, and cytokines that explain the action-reaction of allergies and asthma (Figure 2). In this line, a recent review exposes an interesting concept of “early microbiological programming.” Since life’s early stages are crucial. First contact is in the fetal life with the presence of bacterial DNA in the placenta, amniotic fluid, and meconium (86). The second one is the colonization of the gastrointestinal tract, which takes place from the first days of life till the 3 years of life, then intestinal biodiversity is formed and stabilized in later life (87). Yet, there are crucial factors related to the early colonization of the intestines, airways, and skin. Among them: Early use of antibiotics during pregnancy and in early therapy during newborn stages; Mode of delivery, since the newborn will be colonized by either bacteria from the mother’s skin or vaginal bacteria; Feeding method since breastfeeding promotes colonization with bacteria that reduces the risk of allergies; Newborn and mothers feeding, since unpasteurized milk consumption during pregnancy and infant period may be protective; Early environmental exposure to animals (88).

Throughout infant and adult life, the development of asthma is related to dysbiosis. In patients who have developed the presence of asthma, the microbiological composition of the respiratory tract differs from non-asthmatic. With the isolated bacterial strains of Proteobacteria; Bacteroides predominate in non-asthmatics (89). Yet, there is not enough scientific evidence to support the mechanisms and role between bacterial composition and asthma phenotype (90). The answer as to whether asthma is the cause, or the consequence has only been seen in animal models. In this line, germ-free mice which were exposed to allergens reported a severe allergic reaction. Yet, after bacterial colonization, the allergic reaction was not present (91). Furthermore, the nasal respiratory tract exposed to farm dust results in predominant bacterial colonization of *Acinetobacter lwoffi F78* and *Lactococcus lactis G121*, which are preventive for airway inflammation (92). In human cohort studies, the bacterial colonization of Streptococcus, Haemophilus, and Moraxella in the nasal and lower respiratory tract during the first months of life increased the risk of bronchial asthma, wheezing, and high IgE concentrations (93). However, if this colonization takes place after the first 12 months of life, it no longer increases that risk (94).

In this line, the composition of the lower respiratory and nasopharyngeal tract, as well as the lungs, is affected by intestinal bacteria that are transported through micro-aspiration and bacterial metabolites circulating in the blood (90). One of the most important studies in this regard is the COPSAC2010, in which 690 children were followed from birth up to 5 years of age. The intestinal microbiota and its colonization during the first year of life were associated with the later risk of asthma. Also, low microbial maturity and diversity during the first year of life have an important role in the development of childhood asthma. Thus, the identification of patients, especially in the early stages, who are at high risk for asthma is essential. This may allow for interventions such as manipulation of environmental microbial exposures or the human microbiome. Likewise, the authors suggest potential effects of specific microbial supplementation during the first year of life for children at high risk for developing asthma (95).

## 5. Stress and asthma pathogenesis

Stress is defined as a phylogenetic response developed by an organism, which includes psychophysiological and behavioral modifications, to face the stressor event and ensure survival (96). Regarding physiological modifications, it has been widely described in previous literature how circulating concentrations of pro-inflammatory cytokines showed raised levels related to stress exposure (97), in particular IL-6, IL-1β, IL-2, IL-10, and TNF-α, due to increased production of these cytokines from immune system cells (98), primarily macrophages, and monocytes. Furthermore, previous authors pointed out how this inflammatory response also involves activity from some innate immunity cells, apart from macrophages and monocytes, such as eosinophils, neutrophils, and natural killer cells, as well as activity from adaptive immunity cells, including T and B lymphocytes (99). This inflammatory response has the objective to protect the organism from infections and prevent diseases since the immune system is stimulated. As well, this circumstance also has been described in asthma patients, where some interleukins have been found raised, such as IL6, IL-4, IL-5, and IL-13, secreted from CD4+ lymphocytes, promoting an allergic inflammation which involves a worse prognosis of asthma disease (100, 101).

At a molecular level, it has been widely described how the presence of diverse cytokines could modulate T cell differentiation through different pathways, including signaling cascades. In this way, toll-like receptors (TLRs), and nuclear factor kappa-light-chain-enhancer of activated B cells (NF-κB) are two important factors that are related to asthma pathogenesis, being answerable to inflammation processes, showing complex correlative reactions that involve different molecular changes (102, 103). Regarding asthma, TLR2, TLR4, TLR7, TLR8, and TLR9 polymorphisms have been elucidated by their implication in allergic and asthma pathways (104–106) and are also related to allergic and asthma exacerbations. Then, TLRs identify different pathogen-associated molecular patterns or damage-associated molecular patterns, leading to the stimulation of immune cells and the discharge induction of different pro-inflammatory cytokines and chemokines. Additionally, the activation of TLRs promotes the activity of NF-κB as well as other types of kinases which also enhances the induction of inflammatory cytokines genes. This process is due to the translocation of NF-κB heterodimer to the nucleus, where binding to DNA is possible and consequently activates gene transcription. Finally, NF-κB is also accountable for apoptosis inhibition (107) fact which could compromise cellular renovation (108), harming the cellular cycle.

Related to oxidative stress, numerous research described how the lungs were exposed to a large number of oxidative substances, which may deteriorate lung function. Thus, even oxidative molecules, such as reactive oxygen species (ROS) and reactive nitrogen species (RNS) may be included in normal metabolism processes (109), also airway inflammation and asthma disease could be responsible for the raised production of ROS and RNS, in gran part mediated by eosinophils and neutrophils activity (110), likely having a negative influence in asthma prognosis. Considering these findings, previous researchers suggested the consequent effect of oxidative stress on asthma onset and asthma development (111–113).

Regarding eosinophils, several substances such as major basic protein (MBP), eosinophil peroxidase, eosinophil cationic protein, and numerous cytokines have been identified as cytotoxic and directly related to tissue injury (114, 115). Furthermore, MBP may be related to the liberation of histamine from mast cells, and this discharge of histamine could be liable to neutrophils and macrophage activation, triggering a considerable inflammation event in the airways (116). Additionally, eosinophils may produce several interleukins, including IL-4, IL-6, IL-9, and IL-13, and leukotrienes, all of them related to inflammatory processes (114, 117–119). Finally, recent literature described how eosinophils also could alter airway surroundings, since they have been pointed out as responsible for pro-fibrogenic TGF-β release, producing epithelial and microvascular changes, subepithelial fibrosis, and epithelial damaging (120–122). Moreover, related to molecular pathways previously described, it has been highlighted how eosinophils also present TLRs (123), previously described by their implication in asthma and allergy processes. Thus, added to the fact that also asthma patients showed increased values in eosinophils levels (124), as previous researchers pointed out, eosinophils may be considered an important key factor that could aggravate asthma symptoms and quality of life.

Therefore, regarding the relationship between TLRs, interleukins, and eosinophils, and considering the large number of substances that are contained in eosinophils’ granules and their subsequent effects, it could be determined that these cells constitute a major factor that may be responsible for exacerbation and a potential component in disease progression and symptoms exacerbation. Then, since stress could be contemplated as an eliciting contributor to promoting immune system activation and inflammatory processes (125), which also involves eosinophils activation, it could be considered as an enormous vicious cycle that could compromise asthma disease, being high-stress levels related to a worse asthmatic prognosis (126).

## 6. Metabolic health asthma

Asthma has been largely associated with metabolic diseases which also involved inflammation processes, such as diabetes mellitus type 2, obesity, dyslipidemia, and metabolic syndrome (127–129). Systemic inflammation and oxidative stress processes may be responsible for the connection between asthma and these chronic events (130, 131).

Regarding diabetes, previous authors proposed its relationship with asthma events, since a positive association was found in numerous studies between both diseases, being insulin resistance related to further asthma prevalence (130, 132, 133), probably because both pathological events share common pathways, probably associated with an increase in IL-6 and IL-10 and their pro-inflammatory effects (134). Additionally, this association has been also pointed out as bidirectional, since previous literature described how asthma patients showed a raised risk of diabetes development (135). Furthermore, previous researchers highlighted how diabetes type 2 is more frequent in patients with asthma than in individuals without this pulmonary disease and it was also pointed out that this association could trigger a deficient result in asthma control (128, 136, 137). Moreover, the relationship between asthma and diabetes also has been linked to a larger use of medical sources, including a higher prevalence of hospitalizations, as well as a compromised quality of life, due to the higher incidence of exacerbations and complications present in populations described in recent studies (138–140). One of the physiopathology explanations found by previous researchers which could relate diabetes and a major incidence of comorbidities is that elevated glucose or insulin levels present in the lungs could enhance the growth and differentiation of fibroblasts, harming lung function (141).

Regarding the relationship of asthma and diabetes with other associated pro-inflammatory conditions, previous studies described how asthma and diabetes patients showed an increased risk of developing coronary diseases compared with non-asthmatic patients, suggesting the possibility of a relationship between these three disorders, probably due to the involvement of IL-6 and IL-17 activity, cytokines which developed inflammatory events (137). Furthermore, it has been pointed out by recent literature how asthma could be related to ischemic heart disease (142), since patients in older age (≥ 53 years) showed a significant association between both diseases. Thus, it also could be explained through the contribution of a pro-inflammatory environment which may enhance atherosclerosis (143), negatively affecting vascular modeling and promoting ischemic events. Additionally, previous authors suggested that diabetes type 2, asthma, and obesity also could be related to asthma, since both diabetes and obesity are chronic diseases that also involve systemic inflammation (144, 145). Previous researchers described two different mechanisms which may be responsible for the relationship between asthma, obesity, and diabetes type 2. Primarily, it could be due to the inflammatory conditions, and also it may be explained by the excess fat mass and airway inflammation in asthma which may enhance diabetes development (130).

Regarding metabolic syndrome and dyslipidaemia, it has been largely described how they may be associated with asthma, probably due to IL-6 activity originating from activated macrophages, which promotes systemic and pulmonary modulation, as well as due to the oxidative stress and their effect on airway hyper-responsiveness (146–149). Nevertheless, the key important factor seems to be IL-6, since its levels have been shown raised in patients which presented both poor controlled asthma events and obesity, probably due to its pro-inflammatory effects triggering modulation in systemic and pulmonary inflammation, probably being this IL-6 originated from activated adipose macrophages (150, 151).

Regarding to other metabolic diseases, it has been found how asthma may be related to vitamin D deficiency, since several authors proposed that low levels in vitamin D were associated to current asthma disease (152). These findings were also supported by the fact that a supplementation of vitamin D has been demonstrated as a useful tool which may reduce asthma complications as well as it could enhance asthma control when patients showed a severe low vitamin D baseline levels and intense asthma disease (153–157). These findings may be explained by the fact that raised expression of TNF-α has been associated to low vitamin D levels (158, 159), which may be related to elevated pro-inflammatory conditions, being a deficiency in vitamin D responsible of a pro-inflammatory environment which could compromise development and control in asthma disease (160–162).

Finally, regarding to thyroid metabolism, recent research pointed out how asthma disease could be related to thyroidal dysfunction (163), since asthma may be associated to both hypothyroidism and hyperthyroidism. One more time, the relationship between asthma and thyroidal diseases may be explained by the fact that both share same inflammatory mechanisms, in this case involving a pro-inflammatory subpopulation of CD4+ lymphocytes, Th1 and Th17 pathways (164–166).

It is important to note that the common factors which relate to all these metabolic pathologies are cytokines and oxidative stress, which have been elucidated to have a raised impact that could explain these different pathologies, being all of them interconnected. In this line, obesity has been associated by previous researchers with elevated levels of TLR4, TNF-α, IL-6, C reactive protein, and IL -1β, modulating inflammatory processes which may improve the appearance of other metabolic pathologies such as atherosclerosis, diabetes type 2, and cardiovascular events, being TNF-α and IL-6 those cytokines which seem to take more importance in these processes (105, 141, 167). In this line, also TLR4 has been found raised in areas with atherosclerosis plaque which were relatively close to suffer a separation from vascular wall, as well as it has been found how different polymorphisms in TLR4 may be associated with larger tendency to cardiovascular events (168). Furthermore, TNF-α and IL-6 also have been highlighted to be responsible for the association between obesity and asthma, since they could enhance the production of IL-4 and IL-5, being both found raised in eosinophilic asthma (141, 169). Related to oxidative stress, previous literature pointed out how obese patients produced higher levels of ROS molecules, as well as decreased levels in asthmatic patients (170, 171). Regarding metabolic syndrome, previous authors described how adipose tissues of patients with concomitant obesity and asthma disease produced cytokines, TNF-α, and interleukins, which also promoted systemic inflammatory response (172, 173).

Thus, contemplating findings supported by previous literature, it could be considered that a multifactorial relationship may exist between different metabolic events, all of them mediated by the presence of a pro-inflammatory environment which enhances the development of different pathologies interlinked between them.

## 7. Oral health and asthma

Although there is a relationship between the state of the oral cavity and asthma, the findings are not conclusive. In this line, a study carried out in the United Kingdom, showed that subjects between 11 to 25 years with more plaque and gingivitis presented a greater incidence of asthma (174). Contrary, in another study from Belgium, there was no significant correlation between oral health measures and asthma duration, severity, or medication exposure time in asthmatic children aged 3 to 17 years (175). This study is consequent with the NHANES III study in adolescents aged 13 to 17 years, where neither asthma nor cumulative use of anti-asthmatic medications was significantly associated with gingivitis or periodontitis (176).

Regarding the relationship between the health state of the oral cavity and asthma, authors who affirm its relation suggest that may be due to immunological and inflammatory processes in the patient’s body, the taking of anti-asthmatic medications, especially inhaled ones, or both (177). For example, as in the case of oral candidiasis, which causes would be the immunosuppressive and anti-inflammatory effects of steroids (178). The influence of saliva secretion on periodontal disease is also important when considering the impact of asthma on periodontal disease. The deterioration of the physiology of the periodontal tissues can be the result of a reduced protective effect of saliva, which is combined with an increase in the dry mouth resulting from the process itself. This can cause mouth breathing in the patient, as well as changes in saliva composition and changes in saliva quantity, but can also result from the effects of inhaled corticosteroids. As a result, changes in the number of bacterial and immunological factors, significantly lowering the concentration of immunoglobulin A can be seen (179). Furthermore, changes in the amount of immunoglobulin E and microelements, as calcium and phosphorus, affect susceptibility to further accumulation (178). The increased prevalence of periodontal disease in asthmatic patients, especially those taking inhaled corticosteroids, may also be due to its effect on bone mineral density, including that of the maxilla and mandible (180).

Other diseases such as allergic rhinitis that induce mouth breathing may play an important role in the development of periodontal disease. For example, obstructive sleep apnea (OSA), adenoid and tonsil hypertrophy, and neuromuscular disorders can affect young children (181).

### 7.1. Inhaled corticosteroids and oral health

In this line, inhaled corticosteroids (ICS) are currently the most widely used medications for asthma control. Although they are the most effective long-term maintenance therapy available for mild, moderate, or severe persistent asthma, they present side effects in approximately 10–30% of patients (182). Primary treatment with ICS may favor the development of candidiasis accompanied by hoarseness and cough, dysphonia, xerostomia, changes in the composition and rate of secretion of saliva, changes in the hard tissues of the teeth, periodontitis, and irritation of the oral mucosa and the area around the corners of the mouth (183). Authors suggest that compared to healthy individuals, patients treated with ICS are characterized by: more severe caries (184), more frequent periodontal disease, significantly impaired oral hygiene (185), and malocclusions (186).

### 7.2. Oral microbiome In asthma

Since under healthy conditions, the airway lumen contains mostly air, the availability of nutrients for most microorganisms is relatively limited (187); which could explain the low amount of the bacterial community in healthy subjects. On the other hand, the airways of patients with obstructive airway diseases such as asthma contain a dense growth medium rich in secreted mucus proteins that promote the growth of different types of microorganisms (188). Alterations in the composition and function of the bacterial population appear to contribute to the pathophysiology of asthma. Studies of sputum samples obtained by brushing or nasopharyngeal lavage have reported features of the microbiota associated with asthma. Current evidence presents alterations in the pathophysiology of bronchial bacterial communities in the pathophysiology of asthma in a wide range of clinical presentations of asthma (189).

### 7.3. Asthma and bacterial composition

Durak et.al performed sequencing profiles using the 16S rRNA gene from samples of paired protected bronchial brushings, induced sputum, oral lavage, and nasal brushings from adults with mild atopic asthma, atopy without asthma, and healthy controls. They found that although the nasal microbiota was very distinct from that of the oral or bronchial compartments, the abundance of specific bacterial genera in nasal brushes from asthmatic subjects (Corynebacterium and Moraxella) was associated with asthma and was also associated with markers of systemic inflammation and bronchial. In an association study of atopy, asthma with response to inhaled corticosteroid treatment, the bronchial microbiome differed significantly between groups. Haemophilus, Neisseria, Fusobacterium, and Porphyromonas species and the Sphingomonodaceae family were found mainly in asthmatic subjects. Changes in the microbiota were observed after fluticasone treatment (190).

Denner et al. found significant differences in microbial diversity between brushing and washing samples from asthmatic patients and control subjects. Lactobacillus, Pseudomonas, and Rickettsia species were significantly more frequent in samples from asthmatic patients, while Prevotella, Streptococcus, and Veillonella species were more frequent in brush samples from control subjects. In addition, linear models on brush samples suggest that oral corticosteroid use is an important factor affecting the relative abundance of taxa that were significantly enriched in asthmatic patients. In addition, α bacterial diversity in brush samples from asthmatic patients was correlated with FEV1 and eosinophil ratio in subjects with bronchoalveolar lavage samples (191).

Examining the composition of the airway microbiome in patients with corticosteroid-resistant asthma, 66.6% of subjects with asthma were corticosteroid-resistant (CR), and the remainder were corticosteroid-sensitive CS. The bronchoalveolar lavage microbiome of subjects with CR and CS asthma did not differ in richness, uniformity, diversity, and community composition at the phylum level, but did differ at the gender level (192). In this line, Hilty et al. found that pathogenic proteobacteria, particularly *Haemophilus* spp., were much more frequent in the bronchi of asthmatic adults or patients with COPD than in controls. They found increases in Proteobacteria in asthmatic children. In contrast, Bacteroides, particularly Prevotella spp., were more frequent in healthy controls than in asthmatic adults or children or COPD patients (193).

Hwang et.al found that the bacterial communities associated with worsening of the ACQ score and total leukocytes (mainly eosinophils) in sputum were predominantly Proteobacteria. In contrast, improved/stable ACQ and bronchial epithelial gene expression of FKBP5, an indicator of steroid responsiveness, were correlated with Actinobacteria (194). In a later study, they found that the relative abundance of members of Sphingomonadaceae, Oxalobacteraceae, Comamonadaceae, and other bacterial families was highly correlated with the degree of bronchial hyperresponsiveness in adults (195).

Kloepfer evaluated bacterial presence during peak Rhinovirus (RV) season in children with and without asthma to determine if there is an association between bacterial infection and severity of RV illnesses. Their findings suggest that *S. pneumoniae* and *M. catarrhalis* contribute to the severity of respiratory illnesses, including asthma exacerbations (196).

### 7.4. Asthma and viruses

Viral infections of the respiratory tract are usually self-limited illnesses and common. For patients at risk of asthma, or with existing asthma, viral respiratory tract infections can have a profound effect on disease expression or loss of control. Episodes of wheezing early in life due to rhinoviruses are an important risk factor for diagnosis of asthma at age 6 years. These viral infections are mainly involved in human rhinoviruses, which are associated with exacerbations of asthma. Deficiencies in antiviral activity and airway epithelial barrier integrity could make people with asthma more prone to serious viral lower respiratory tract infections and thus increase the risk of exacerbation. Given the effect of respiratory viruses on many aspects of asthma, efforts to understand the mechanisms and risk factors by which these airway infections are associated with changes in airway pathophysiology are the first step toward better treatment (197).

### 7.5. Asthma and candidiasis

Oropharyngeal candidiasis is common in patients using corticosteroids, with a direct correlation between high doses and exposure time. Oral candidiasis includes symptoms such as an unpleasant sensation in the throat and pharyngodynia (198). It is mainly due to the immunosuppressive effects of ICS (178) and decreased concentrations of salivary IgA and histatin (198). Nearly 20% of the inhaled dose reaches the lungs, with most remaining in the oropharynx. In addition, many dry powder inhalers (DPIs) contain lactose monohydrate as a carrier, which can promote Candida growth and worsen *Candida*. Beta 2 agonists may also contribute to a higher incidence of candidiasis by reducing the concentration of saliva (177).

Fukushima et.al found that the amount of *Candida* spp. was significantly higher in asthmatic patients taking inhaled steroids (fluticasone and beclometasone) compared to those not taking them. It was also significantly higher in patients with oral symptoms than in asymptomatic patients and significantly higher in asthmatic patients treated with fluticasone than in those treated with beclomethasone. The presence of *Candida* was positively correlated with the dose of fluticasone. Also, gargling with amphotericin solution reduced the number of Candida and improved symptoms (198).

Kurt et al. analyzed the frequency of oropharyngeal candidiasis in asthmatic patients using fluticasone propionate (FP) as a dry powder inhaler. The frequency of Candida colonization was higher in the group of FP users than in asthmatics without ICS use. The most effective variables in preventing the occurrence of Candida colonization were throat washing by patients and duration of ICS use greater than 12 months. In patients using ICS, the most important determinants of colonization were not washing the throat regularly and the duration of ICS use for more than 12 months or more (199).

To reduce the incidence of oral candidiasis, the following preventive measures can be taken: Rinse the mouth and use a spacer device, administer topical antifungals (eg, nystatin), use sialagogue medications in patients with the low salivary flow, chew sugarless gum, gargling with a 1:50 diluted amphotericin solution rather than just gargling with water (198).

However, although most of the studies described above have shown a relative frequency of the presence of oropharyngeal Candida in asthma diagnosed adult and child patients with compared to healthy subjects (200); There is no proven causal relationship of disease with this type of microorganism, but there is a higher susceptibility of people with asthma to the growth of oropharyngeal candidiasis. In this line, it was recommended that patients who use inhaled corticosteroids be informed at the start of treatment of the side effects of these drugs to adjust the dose (201).

## 8. Physical activity interventions

Physical activity is an important factor for correct development and correct organism systems. We are animals made by and for movement, we need movement so that the powerful endocrine organ that is our skeletal muscle works and can produce the basic cytokines for the regulation of other organic processes in the rest of the body systems (202, 203). The endocrine functions of muscle myokines are the basis for organism regulation and have an important role in inflammation processes (204). In this line, physical activity is shown as an important intervention tool in asthma.

Previous studies have reported that low levels of physical activity are associated with negative health consequences (i.e., poorer respiratory functioning: lower PEF and FEV1), higher asthma symptoms, poorer quality of life, a decrease in physical and mental health, and an increment in the use of health care resources (higher physician visits and hospital stays) (205–209). Moreover, it has been reported a link between the risk of asthma prevalence and asthma severity and physical inactivity (210). For this reason, the inclusion of physical activity as a non-pharmacological coadjutant treatment is well established due to its beneficial impact on patients with asthma (211). In this way, many guidelines published by international associations have recommended an increase in physical activity to provide health benefits for chronic diseases, including asthma (212, 213). Despite the well-established benefits of exercise in patients with asthma, they are less likely to engage in physical activity than people without asthma (205). In addition, individuals with asthma prefer to perform low-intensity exercise than high-intensity activities when they make physical activity (214).

One possible reason that explains this paradox may be associated with the fact that exercise can trigger asthma symptoms and exacerbations leading to an avoidance of physical activity in individuals with asthma (211, 215). In this way, previous studies reported an increase in asthma symptoms as a result of exercise in a range from 40 to 90% in patients with asthma (205, 216, 217). However, some possible aspects can help the patient to have a balance between exercise benefits and asthma attacks trigged by exercise. The education of the patient to choose the types of exercise that are less likely to cause asthma or the inclusion of warm-up before exercise can improve the management of asthma attacks associated to exercise (218).

During the last years, some systematic reviews that analyze the effect of exercise on asthma symptoms (211, 219–222) have been published reporting controversial results. Regarding the evidence of physical activity on lung function parameters, a previous review reported positive effects of physical training in patients with asthma but unrelated to effects on lung function (219). However, another recently systematic review (211) concluded that physical activity is associated with improvements in lung function (e.g., peak expiratory flow, forced expiratory volume in the first second of expiration, forced expiratory flow at 25% of forced vital capacity). In this way, some of the randomized controlled trial studies in this area (223–227) indicate that aerobic exercise produces a physiological improvement in lung functioning and a decrease in asthma exacerbations. The well-known bronchodilator effect of aerobic exercise associated with lung expansion (228) produces that this type of exercise was the most recommended activity in patients with asthma.

The improvement in quality of life in patients with asthma is one of the goals of any treatment. Most of the experimental studies (229–232) and systematic reviews (211, 219) that analyze the effect of aerobic exercise on health-related quality of life outcomes found an improvement in health-related quality of life. Moreover, other studies focused on the effect of exercise training on serologic inflammatory markers. In this way, inflammatory markers are recently considered a therapy target in patients with asthma because of a relationship between severe asthma and the values of inflammatory markers such as IL-6 (233). Remarkably, previous studies found a positive effect of physical activity on serologic inflammatory markers, decreasing IL-4 and TNFα (231, 232).

Asthma control is a key component for the patient (234) and it is usually reported by a questionnaire (i.e., asthma control questionnaire (ACQ)), the number of asthma exacerbations, using daily diary symptoms, or the number of reported bronchodilator inhaler use, among others. Remarkably, regular exercise has also demonstrated its effectiveness in asthma control. In this way, some studies found an improvement in ACQ and asthma exacerbations using moderate aerobic training (232) or high-intensity training (235, 236). Moreover, previous studies reported a decrease in the number of bronchodilator inhaler use (230) and an improvement in the symptoms reported using a daily diary (237) after aerobic exercise.

Lastly, there is also some evidence that exercise improves other physiological and psychological variables in individuals with asthma. Noteworthy, exercise improved autonomic modulation measured by heart rate variability in patients with asthma. In this way, a relationship between poorer heart rate variability and mild–moderate diagnosis of asthma (REF) (238). On the other hand, physical activity is associated with improvements in mental health (i.e anxiety and depression) in patients with asthma (205). This fact may be particularly interesting in this population because anxiety negatively impacts asthma (239).

Regarding the exercise dosage, little is known about the most efficient training characteristics (volume, intensity, frequency, or density) to provide health improvements in patients with asthma. In addition, there is little knowledge about the type of exercise to recommend to individuals with asthma. Although individualization training and adherence to the program are key factors to obtaining a successful exercise therapy, a total of studies which are found health benefits used aerobic exercise (209, 237, 240–242). In this way, the existing literature has examined the impact of some modalities of aerobic training such as walking, stationary cycling, or swimming, isolated or combined with strength exercises such as calisthenic exercises or circuit training. This last type of exercise, circuit training, is an interesting tool to develop cardiorespiratory fitness and strength concomitantly and is usually effectively applied in some populations like young and older and chronic disease patients (243–245). On the other hand, the most common duration of the training session is 30–45 min with a weekly frequency of 2–3 days per week at moderate intensity (60–70% of maximum oxygen uptake) and lasting from 6 to 12 weeks. In addition, the exercise intervention must be programed and supervised by health or sports professionals to obtain the maximal benefit because a previous self-guided intervention demonstrated no improvement in lung function (246). Despite the limitation of the low evidence of the optimal exercise dosage which provides the most benefit to individuals with asthma, it is demonstrated the beneficial effect of exercise in patients with asthma and the importance of encouraging them to adhere to exercise programs and to perform exercise regularly.

## 9. Physiotherapy interventions

Evidence showed the effectiveness of non-pharmacological treatment of asthma as a coadjuvant of meditation in individuals with asthma. These non-medical treatments include the aforementioned physical therapy and exercise and various physiotherapy techniques including breathing exercises and inspiratory muscle training, among others (247, 248). In this way, a reduction of the inflammation of the airway, an improvement in the patency of the bronchioles, and an increase in the strength values of the respiratory muscles have been found after physical therapy treatment in individuals with asthma, improving their lung function (249).

Remarkably, patients with asthma have abnormal or dysfunctional breathing patterns (250). Therefore, the target of some breathing therapies applied is focused on breathing retraining, which is aimed at reducing hyperventilation and hyperinflation, slowing the respiratory rate, and prolonging the expiratory phase, and enhancing abdominal and diaphragmatic breathing, encouraging nasal breathing (247). In this way, international guidelines for the physiotherapeutic management of asthma recommend breathing exercises in individuals with asthma due to the level of evidence of this therapy to increase asthma control and the quality of life of the patients (251). Some techniques, such as the Papworth method, the Buteyko breathing technique, and Yoga have been demonstrated their effectiveness (252) to produce the rehabilitation of the breathing pattern, showing an improvement in quality of life, reducing symptoms, hyperventilation, anxiety, and depression, lowering the respiratory rate and the use of medication, but not affection lung function (247).

The most common symptoms in individuals with asthma are cough, shortness of breathing duration, wheezing, and chest tightness (169). This chest tightness may be due in part to a dysfunction of the skeletal muscle of the chest wall and the shoulder girdle, contributing to the disease manifestations (253). For this reason, the use of manual therapy for posture correction and muscular function improvement may improve asthma symptoms. In this way, the application of a concomitant strength and stretching technique consisting of an eccentric stretching of the arm, shoulder, and chest while the patient lying in a supine position (four treatments over 47 ± 21 days) promotes a clinically and statistically significant improvement in the asthma control (253). However, a previous Cochrane review about the effect of manual therapy on asthma, including manipulation, mobilization, massage, chest percussion, shaking, and vibration found no evidence of effectiveness (254).

The capacity of the respiratory muscle to generate tension in asthmatic patients is reduced (255). The increase in the cross-sectional area of the inspiratory muscle could improve the functional weakening induced by asthma (255). In this way, the inspiratory muscle can be trained using an external resistive device, in the technique called Inspiratory muscle training (IMT). This procedure can improve the endurance and the strength of the inspiratory muscles (diaphragm and accessory inspiratory muscles) (256). Regarding the evidence about IMT and respiratory benefits in asthma patients, a previous Cochrane review (255), limited by the small number of studies, it is concluded that there is no conclusive evidence in this review to support or refute inspiratory muscle training for asthma. More recently, a systematic review and meta-analysis (257) including more randomized controlled trials (n = 6) concluded that IMT may benefit the treatment of asthma, increasing maximal inspiratory pressure and decreasing dyspnea perception. In this way, some randomized controlled trials found that IMT is an effective technique to enhance respiratory muscle strength, exercise capacity, quality of life, and daily living activities and reduce the perception of dyspnoea and fatigue in individuals with asthma 258,259. Notably, treatment dosage is heterogeneous in the literature. In this way, studies used a frequency of once or twice daily, five-six days per week, for at least 3 weeks, performing 30 breaths or 30 min of training at 15–60% of the maximum inspiratory pressure (258–260). Thus, the IMT may be included in the non-pharmacological treatment in patients with asthma due to the health benefits demonstrated.

In summary, the evidence of physical therapy for asthma is limited by the small number of trials. Therefore, the results of some physical therapies should be used by caution and more well-conducted randomized controlled trials are needed to determine the level of evidence of some of these techniques. However, the current evidence shows that breathing exercises and inspiratory muscle training are promising techniques to improve asthma symptoms.

## 10. Nutritional interventions cause

In recent decades the prevalence of asthma has continued to increase as Western dietary patterns have taken hold (261). Data suggest that diets that prioritize plant-based foods and limit consumption of animal products along with weight control may mediate cytokine release, free radical damage, and immune responses in the development and progression of asthma (261). In this context, the prevalence of asthma has been increasing in parallel with the westernization of dietary patterns (262). Thus, some authors have suggested that this westernization of dietary patterns in Latin American countries could play an important role in increasing the prevalence of asthma in their citizens (263).

In this regard, it has been shown that a high fat intake and low fiber intake, a pattern typical of the Western diet, has been associated with airway inflammation, through an increase in eosinophilia, and a worsening of lung function, through FEV1, in asthmatic patients (264). Furthermore, saturated fat intake was positively associated with a higher percentage of eosinophils in sputum which is correlated with asthma severity and lung function impairment (265, 266). Likewise, it has been found that a high-fat diet (> 60%) led to increased airway hyperresponsiveness through increased cytokine production in the lung (267). Even an improvement in peak expiratory flow rate has been observed in 22 children who for 8 weeks did not drink milk or eggs (268).

Among the foods of animal origin that have been most studied for asthma are dairy products. Thus, frequent consumption of dairy products seems to have a positive association with the probability of developing asthma in children (269). Along these lines, a positive association has been observed between the consumption of ricotta cheese and concurrent asthma and low-fat cheese with physician-diagnosed asthma, and both types of cheese with bronchial hyperreactivity in children (270).

On the other hand, although the consumption of 475 mL of skimmed or whole milk did not affect FEV1 and forced expiratory flow at 50% of vital capacity measured every 30 min for 3 h, whole milk did cause a progressive deterioration in the capacity for pulmonary diffusion of carbon monoxide in asthmatic patients (271). Similarly, acute ingestion of 300 mL of cow’s milk caused 8 of the 20 asthmatic patients studied to perceive asthma symptoms although dairy products do not have a specific bronchoconstrictor effect in asthmatic patients, regardless of their perception (272). Finally, 10 g of powdered whole cow’s milk caused a slight decrease, with little clinical significance, in FEV1 (3.3%) and FEV1/FVC (2.7%) in 25 adult patients with asthma (273). In this regard, the use of conventional spirometry to detect dairy-induced pulmonary changes may lack the necessary sensitivity (261).

Although the mechanisms by which dairy products may be involved in the development and progression of asthma are unclear, the cause is likely related to responses to milk proteins or lipids (271). In this regard, it is recommended that saturated fat intake be reduced to <7–10%. However, it should be well ensured that patients with asthma do not unnecessarily restrict dairy intake by risking the development of nutritional deficiencies despite some associations between milk consumption and some asthma symptoms. To this end, larger sample studies could better clarify the possible connection between dairy products and clinical symptoms (261).

On the other hand, diets that prioritize the consumption of fruits and vegetables and whole grains, as opposed to lower consumption of meats and high-fat dairy products, have been associated with a lower risk of asthma. Thus, a lower probability of asthma diagnosis has been reported in children who consume large amounts of fruits, vegetables, legumes, cereals, pasta, rice, and potatoes and little meat (274, 275).

Similarly, Mediterranean-style dietary patterns that prioritize plant-based products have also been associated with reduced asthma symptoms in asthmatic children (275–277). Thus, greater adherence to the Mediterranean diet was associated with greater lung function and lung function (FEV1 and FVC) (278). Similarly, after a one-year Mediterranean diet program, the intensity of asthma attacks, infections, hospital admissions, and medication in asthmatic children was improved (279). Even the protective effect of the Mediterranean diet on wheezing in children has been shown (276).

Another type of diet that prioritizes the consumption of fruits, vegetables, cereals, and legumes and in this case eliminates foods of animal origin is the vegan diet. In this regard, clinical improvements in vital capacity, FEV1, and physical work capacity have been observed after asthmatic patients adopted a vegan diet for 1 year (280). These results together with those shown by the Mediterranean-style diet suggest that a plant-based diet provides a potential treatment option for asthmatic patients.

The results of this type of diet indicate that high consumption of fruits and vegetables was associated with a lower risk of developing asthma and a lower incidence of wheezing in both children and adults (281). Likewise, fruits and vegetables seem to make asthma symptoms more bearable. Thus, it has been shown that long-term fruit intake (between 2 to 8 years) was inversely associated with asthma symptoms and with sensitization to inhaled allergens (282). Specifically, the intake of fruit 3 times per week and vegetables was inversely associated with asthmatic wheezing and severe asthma symptoms in children aged 6–7 years. Adolescent and adult patients also experienced similar protective effects with fruit and vegetable intake (283). In addition, intake of 5 servings of vegetables and 2 servings of fruit per day for 14 days showed a higher predicted percentage of FEV1 and forced vital capacity (FVC) than those who consumed a diet with 2 servings of vegetables and 1 serving of fruit (284).

Although in many cases the studies do not specify whether the vegetables were raw or cooked, Iikura et al. (285) suggested that the anti-inflammatory effect of the flavonoids in vegetables is lost upon heating, which would explain the positive association observed by these investigators between 5 or more servings per week of raw vegetables and well-controlled asthma. Similarly, high citrus intake (>46.3 g/d) was associated with a lower risk of asthma symptoms (286). In this sense, everything seems to indicate that a diet high in plant foods appears to reduce pro-inflammatory molecules and increase anti-inflammatory markers (284, 287). In addition, other nutrients included in these foods such as unsaturated fatty acids and antioxidants seem to attenuate the condition and inflammation produced by the systemic inflammatory response (288, 289). However, controlled studies are needed to further explore the relationship between dietary patterns and asthma symptoms.

## 11. Ergogenic interventions

### 11.1. Dietary antioxidants

As indicated in a previous section, it has been suggested that antioxidants present in fruits and vegetables may confer a protective effect against asthma (290–292). Antioxidants prevent, intercept, and repair the effects of oxidation and cell damage. Although studies with antioxidant supplementation are limited, there is sufficient evidence to consider these nutrients essential in the treatment and prevention of asthma. Dietary antioxidants include vitamins A, E, and C, ubiquinone, flavonoids, and selenium (293).

Vitamin A and its precursor, beta-carotene, are known to have effects on mucosal surface integrity and stability and may enhance antioxidant defense systems against oxidative stress (294). Other dietary carotenoids (including a-carotene, b-cryptoxanthin, lutein/zeaxanthin, and lycopene) are also associated with improvements in lung function (292). In this regard, improved lung function has been observed in older women who had higher serum a-carotene and b-carotene levels (261). On the other hand, a negative association has been shown between overall dietary vitamin A intake and the odds of developing asthma and severe asthma, although the results for wheezing were less consistent (295). In addition, vitamin A intakes in people with asthma were found to be approximately 50% below the recommended daily intakes, being lower in people with severe asthma than in those with less severe asthma. While the results for the different forms of vitamin A (retinol, α-carotene, total carotenoids, and carotene) showed no significant differences, there was a trend that increased serum levels of β-carotene were associated with a reduced likelihood of asthma (296). Epidemiological evidence suggests that vitamin A is associated with asthma. Further research is needed to explain the associations observed using well-designed randomized controlled trials of vitamin supplementation in asthma.

Vitamin E, composed of tocopherols and tocotrienols, is known to have several cellular effects such as modulating the synthesis of pro-inflammatory molecules and the response to oxidative stress (297). In this line, plasma *α-tocopherol* is associated with lung function in patients with and without asthma (298). Similarly, vitamin E biochemical levels are lower in adults with asthma than in those without the disease, while higher dietary vitamin E intake is associated with a lower incidence of asthma (299). On the other hand, A combination of antioxidant supplements including vitamin E has been shown to reduce ozone-induced bronchoconstriction in people with and without diagnosed asthma (261). Therefore, it seems reasonable to think that vitamin E supplementation, at least in asthma patients with low serum vitamin E levels, could be an effective asthma treatment.

Vitamin C in addition to promoting hydration of airway surfaces acts as a cofactor in several immunomodulatory regulatory enzymes (300). Vitamin C is also involved in the metabolism of histamine and prostaglandins, which are involved in bronchoconstriction (301). Thus, there is strong evidence that, in some conditions, vitamin C can reduce exercise-induced bronchoconstriction (301, 302). In addition, studies providing direct information on vitamin C and common cold-induced asthma (302) have been found to support the hypothesis that vitamin C may benefit some people suffering from asthma exacerbation during colds (303). Finally, it should be noted that although vitamin C may not be effective for patients with permanent stable asthma, it may have a beneficial effect on lung function in some asthmatics under certain forms of acute stress, such as when they are under intense physical activity or suffer from a viral respiratory tract infection (301, 302, 304, 305). However, there was evidence of an increased likelihood of asthma associated with lower and average levels of both serum and vitamin C intake. These findings were generally supported by findings of wheezing and airway reactivity; however, associations with asthma severity were inconclusive (295). Based on all this evidence, vitamin C should be included in the list of possible supplements for asthma.

The flavonoids have been recognized as antioxidants and metal chelators (295). Flavonoids also exhibit anti-inflammatory and anti-allergic activities (306). Flavonoids may be part of the positive effect that fruit consumption has on asthma (307). Thus, kaempferol, a flavonoid found in apples and many berries, has been shown to alleviate airway inflammation and act as a therapeutic agent for asthmatics (308) by inhibiting IL-8 and oxidative stress induced by LPS and H2O2 in airway epithelial cells (309). Another flavonoid, quercetin, commonly found in fruits and vegetables, seeds, nuts, tea, and wine, has strong effects on reactive oxygen species metabolism and cell apoptosis (310). While no studies with a direct link to asthma have been located, it has been observed that oral quercetin revived lung antioxidant defense mechanisms along with restoration of lung pathology (311) and was able to alleviate radiation-induced oxidative stress, pneumonitis, and fibrosis (312), as well as LPS-induced lung injury (313). Considering these pathways, flavonoids may be therapeutic candidates for asthma, not only for their protective effects as antioxidants but also for their modulatory effects on cell signaling cascades related to inflammation and lung pathology (103).

Selenium is a cofactor of the antioxidant enzyme glutathione peroxidase which prevents lipid peroxidation. In this regard, reduced glutathione peroxidase activity has been observed in asthmatic patients together with a lower concentration of selenium in plasma and whole blood compared to their non-asthmatic counterparts (314). Thus, studies suggest that the consumption of dietary sources of these antioxidants may protect against the effects of oxidants and prevent inflammation (315).

These nutritional antioxidants can be obtained through a plant-based diet and present a potential method of treating asthma symptoms but could be used through nutritional supplementation if their serum and/or nutritional values are not within the appropriate range.

### 11.2. Omega-3 polyunsaturated fatty acids

It has been suggested that while some types of polyunsaturated fats may play a protective role, saturated fats may induce inflammation. In this respect, it has been suggested that supplementation with certain types of polyunsaturated fats may be beneficial for the prevention and/or treatment of asthma.

In this regard, it has been observed that an increase in the consumption of n-6 polyunsaturated fatty acids (PUFA) together with a decrease in the consumption of n-3 polyunsaturated fatty acids (PUFA) led to an increase in childhood asthma (316, 317). One proposed mechanism for this correlation is that increased consumption of linoleic acid (n-6 PUFA) increases proinflammatory mediators through its conversion to arachidonic acid (318–320). In contrast, n-3 PUFAs inhibit the conversion of linoleic acid to arachidonic acid, thus bypassing this inflammatory pathway (319). *α-linolenic acid* (n-3 PUFA) is converted to eicosapentaenoic acid, which can competitively inhibit arachidonic acid metabolism and thus bypass the inflammatory cascade (318). In this regard, it has been shown that higher n-3 PUFA intake was associated with better asthma control, lower doses of inhaled corticosteroid medication, and lower systemic inflammatory markers, suggesting that n-3 PUFA may have a role in the treatment of asthma (321). However, intervention studies are needed to confirm this hypothesis, especially in specific subpopulations such as obese people with asthma, who may benefit more from this type of dietary intervention.

Therefore, beyond promoting a higher dietary intake of n-3 PUFAs, which can be obtained from green leafy vegetables, flaxseeds, and nuts, and reducing the intake of n-6 PUFAs, which are commonly found in vegetable oils and animal fats, supplementation with n-3 PUFAs could be considered with low plasma levels and/or low intakes of n-3 PUFAs.

### 11.3. Vitamin D

The active form of vitamin D [1,25(OH)2 D] plays a key role in innate and adaptive immunity, and thus may defend against respiratory infections and inflammatory diseases, including asthma (322, 323). Thus, vitamin D insufficiency may also stimulate inflammatory responses to non-pathogenic bacteria in the gut, thereby mediating asthma and immune function through the role of vitamin D in the gut microbiome (324).

Epidemiological studies have suggested a relationship between the prevalence of asthma and vitamin D insufficiency and deficiency in children, showing that a deficiency or insufficiency of vitamin D was significantly higher in asthmatic patients than in controls (325). In this line, vitamin D deficiency was found to be the strongest of the 8 possible predictors of asthma (326). Thus, children with vitamin D deficiency (< 20 ng/mL 25-hydroxyvitamin D) were 5 times more likely to have asthma, while those who had insufficient levels of vitamin D (< 30 ng/mL 25- hydroxyvitamin D) were 3 times more likely (327). In addition, vitamin D insufficiency is also related to asthma severity 126,127 and an increased risk of hospitalization or emergency room visits (328, 329). In this regard, a meta-analysis elucidated that vitamin D supplementation played a role in reducing the rate of asthma exacerbation, particularly in patients with vitamin D insufficiency. In addition, it also had an improvement in FEV1% in air-limited patients with vitamin D insufficiency (157). Although larger, well-designed studies are needed to evaluate the role of vitamin D in asthma symptomatology and prevention, vitamin D supplementation represents a potentially low-cost, low-risk therapeutic option to treat and control asthma.

## 12. Psychological interventions

Chronic diseases such as asthma produce important emotional dysregulation in the patients who suffer from it (330). However, historically, this disease has been treated from a purely physiological approach, seeking that the patient can control the inflammatory respiratory symptoms and airway obstruction since it causes great discomfort and a high deterioration in the quality of life (331).

However, for some years now it has been possible to assess the presence of other factors that generate great discomfort in patients with asthma, and among the most important is the symptomatology associated with mood disorders such as anxiety and depression, whose prevalence in this population is very high (332). Studies in this line of work indicate that people with asthma are up to six times more likely to suffer from mood disorders during their lifetime (333). This can probably be explained by the high comorbidity of respiratory disease and by the high mortality rate, around 10% of patients diagnosed with chronic asthma (334).

Particularly with this profile, it is important that asthmatic patients can obtain psychological help that allows them to control the disease, generate a healthy bond of attachment to the pharmacological treatment, and adequate emotional strategies to improve the quality of life from a biopsychosocial perspective (335).

Recent studies confirm the presence of psychological pathologies that coexist with the chronic and degenerative disease of asthma (336). Particularly, in those patients with asthma whose control is complicated. Interestingly, this difficulty in controlling the disease causes anxious-depressive symptoms which, in turn, trigger more severe asthmatic crises (334). It should also be taken into consideration that breathing disorders have an important effect on the psychological state of the person, as he or she goes through moments in which difficulty in breathing can lead to thoughts of helplessness, a feeling of suffocation, and, ultimately, a sensation of imminent death (337).

This feeling, which inevitably produces high tension, causes anxiety that will activate the sympathetic nervous system, and with it, the well-known reactions that accompany this activation such as sweating, increased heart rate, and even pressure in the chest area that increases the feeling of not being able to breathe (338). But there is also an excess of cortisol in the prefrontal regions of the brain, which will prevent proper decision-making, or facilitate the presence of negative and disorganized thoughts that prevent remaining calm (339).

Psychological factors are an essential part of the genesis and progression of asthma (340). This can be seen, for example, in hospital emergency departments, and it has been verified that asthmatic patients who also have a mood pathology come more frequently to these departments and present a greater severity in the symptoms of the disease (341). In addition, it has been observed that asthma patients with comorbidity with depression have higher rates of sudden death, which could be explained by greater difficulty in adhering to pharmacological treatment (342). However, asthmatic patients treated in mental health services, who follow pharmacological treatment with tricyclic antidepressants and cognitive-behavioral psychological therapy, show a notable improvement in asthma symptomatology, a reduction in the use of inhalers, and a significant improvement in quality of life (343).

Therefore, in the psychological intervention with asthmatic patients, it is essential to consider three fundamental aspects that must be addressed. On the one hand, the emotional component: emotions will have an impact on the evolution of the disease since negative emotions such as anger can trigger asthma attacks or re an anxious state of hyperventilation that favors shortness of breath (344).

Secondly, the cognitive variants: the patient must be able to identify the signs that will predict the onset of an asthmatic crisis, to be able to take measures and reverse the process. If the person can identify the risk factors that are a warning, he/she will be able to train strategies and skills focused on controlling these aspects (345). This will lead to a better understanding of the disease and a greater sense of control on the part of the patient, which will immediately lead to a better relationship with his disease and will also improve his relationships with other people (346).

Finally, the behavioral aspects that should be worked on in psychological therapy include the promotion of positive behaviors of acceptance of the disease, adherence to treatment, and also favoring habits such as hobbies, sports, or any activity that can be a way of dealing with the symptoms of asthma positively and that allows the incorporation of tools that help the patient to understand the disease and the best way to live with it throughout the life cycle (347).

It is also important to consider that asthma often presents comorbidity with other pathologies such as hypothyroidism, diabetes, hypertension, or gastritis, among others (348). Suffering from these diseases may mean that the person will need a therapy that addresses in detail how they impact day-to-day life, what is the level of discomfort involved and what is the functionality that the patient can achieve in the situation in which he/she arrives at the psychologist’s office (349).

With all this, studies have been carried out along the lines of applying a cognitive behavioral therapy intervention in this type of patient (350). In these studies, a previous evaluation has been carried out in which high scores were obtained in anxiety measurement instruments such as the Hospital Anxiety and Depression Scale (HADS) and an interview for patients with asthma adapted to include general patient data (351). A programmed intervention was carried out that consisted of a total of 8 sessions, from the application of guided imagery with instructions on the behaviors to be treated, to the last follow-up and control session (352). After this program, more than 80% of the treated patients presented lower scores on the anxiety scales applied at the end of the program (353).

In this line, we found studies with severe asthmatic patients whose design is based on a non-pharmacological clinical trial whose main objective is to evaluate the efficacy of the emotional and cognitive situation of the patients (354). Control groups were used to observe differences in outcomes (355). Anxiety levels were measured with STAI-E and STAI-R, depression levels with the BECK Depression Inventory (BDI) as well as other levels that may impact lung capacity and alteration of symptom severity, such as hyperventilation syndrome (356). Significant relationships were found between asthmatic symptomatology and emotional disorders (357).

Another study by Ciprandi et al., found a relationship between 11% of asthmatic patients with depression and almost 37% with anxiety, measured with the HADS questionnaire. Another study, SARP (Severe Asthma Research Population) showed that asthmatic patients are up to 2.4 more likely to have anxiety, depression, insomnia, and other symptoms associated with mood disorders (358, 359). Previous studies shown that high levels of anxiety will favor a worse control of respiratory functions and worse control of asthmatic symptomatology (360).

## 13. Practical applications

As the main practical application of the present study, we can highlight.

- Excessive hygiene and the absence of germs together with early vaccinations and increasing levels of contamination facilitate the early onset of allergies and asthma in children.
- The early exposition to environmental outdoor and indoor microbiome may play a significant role in asthma pathogenesis during childhood.
- Specific microbial supplementation in the first year of life for children could reduce the risk of developing asthma.
- Since stress could be contemplated as an eliciting contributor to promoting immune system activation and inflammatory processes, it could be considered an important factor related to a worse asthmatic prognosis.
- Metabolic events mediated by the presence of a pro-inflammatory environment could enhance the development of asthma.
- Taking anti-asthmatic medications, especially in-haled ones, or both can affect the health of the oral cavity causing a greater number of inflammatory processes in the body of asthmatic patients.
- Despite the benefits shown by aerobic training in asthma treatment, encouraging patients to adhere to exercise programs and to perform exercise regularly has been proposed as a key factor.
- Breathing exercises and inspiratory muscle training are promising techniques to improve asthma symptoms.
- Long-term fruit intake and vegetables (between 2 to 8 years, 3 times per week) have been inversely associated with asthma symptoms development and with sensitization to inhaled allergens.
- Nutritional antioxidants obtained through a plant-based diet could be used as nutritional supplementation for treating asthma symptoms.
- Asthmatic patients who follow pharmacological treatment with tricyclic antidepressants and cognitive-behavioral psychological therapy, show a notable improvement in asthma symptomatology.

## 14. Conclusion

Asthma is one of the most common chronic respiratory diseases worldwide and often begins in childhood but can also develop during adulthood. Indeed, asthma is the consequence of a complex interaction between different factors in very specific periods of life. In this line, early exposure to microbial environments, as well as microbial diversity in the gut, may reduce the risk of later stages of life disease due to a strengthened immune system. In addition, stress episodes or taking inhaled medications can increase the inflammation-causing shortness of breath, which exacerbates asthma symptoms. Physical activity has been focused on the benefits that aerobic training can provide, while physiotherapy interventions recommend breathing exercises to improve the quality of life of patients. Moreover, nutritional interventions are targeted on implement diets that prioritize the consumption of fruits and vegetables and supplementation with antioxidants. Furthermore, psychological interventions have been proposed as an essential non-pharmacological tool to reduce the emotional problems associated with asthma.

## Author contributions

VC-S: conceptualization, supervision and project administration. JT-A: methodology. VC-S, JM-A, DR-C, AB-V, IM-G, ENJ, LR-F, RY-S, and JT-A: investigation, writing–original draft preparation, writing–review and editing, and visualization. All authors contributed to the article and approved the submitted version.

## References

[1] Bousquet J Mantzouranis E Cruz AA Aït-Khaled N Baena-Cagnani CE Bleecker ER. Uniform definition of Asthma severity, control, and exacerbations: document presented for the World Health Organization consultation on severe Asthma. J Allergy Clin Immunol U S A. (2010) 126:926–38. doi: 10.1016/j.jaci.2010.07.019, PMID: 20926125

[2] GBD 2019 Diseases and Injuries Collaborators. Global burden of 369 diseases and injuries in 204 countries and territories, 1990–2019: A systematic analysis for the global burden of disease study 2019. Lancet. (2020) 396:1204–22. doi: 10.1016/S0140-6736(20)30925-9, PMID: 33069326 PMC7567026

[3] Eagan TML Brøgger JC Eide GE Bakke PS. The incidence of adult Asthma: a review. Int J Tuberc Lung Dis. (2005) 9:603–12. PMID: 15971386

[4] Torén K Gislason T Omenaas E Jögi R Forsberg B Nyström L. A prospective study of Asthma incidence and its predictors: the RHINE study. Eur Respir J. (2004) 24:942–6. doi: 10.1183/09031936.04.00044804, PMID: 15572536

[5] To, T Stanojevic S Moores G Gershon AS Bateman ED Cruz AA. Global Asthma prevalence in adults: findings from the cross-sectional world health survey. BMC Public Health. (2012) 12:204. doi: 10.1186/1471-2458-12-204, PMID: 22429515 PMC3353191

[6] World Health Organization. *Assessing National Capacity for the prevention and control of noncommunicable diseases: report of the 2019 Global Survey*. (2020).

[7] Clemente-Suárez VJ Navarro-Jiménez E Simón-Sanjurjo JA Beltran-Velasco AI Laborde-Cárdenas CC Benitez-Agudelo JC. Mis--dis information in COVID-19 health crisis: a narrative review. Int J Environ Res Public Health. (2022) 1:5321. PMID: 35564714 10.3390/ijerph19095321 PMC9101334

[8] Clemente-Suárez VJ Ramos-Campo DJ Mielgo-Ayuso J Dalamitros AA Nikolaidis PA Hormeño-Holgado A. Nutrition in the actual COVID-19 pandemic. A narrative review. Nutrients. (2021) 13:1924. doi: 10.3390/nu13061924, PMID: 34205138 PMC8228835

[9] Clemente-Suárez VJ Martínez-González MB Benitez-Agudelo JC Navarro-Jiménez E Beltran-Velasco AI Ruisoto P. The impact of the COVID-19 pandemic on mental disorders. A critical review. Int J Environ Res Public Health. (2021) 18:10041. doi: 10.3390/ijerph181910041, PMID: 34639341 PMC8507604

[10] Clemente-Suárez VJ Navarro-Jiménez E Ruisoto P Dalamitros AA Beltran-Velasco AI Hormeño-Holgado A. Performance of fuzzy multi-criteria decision analysis of emergency system in COVID-19 pandemic. An extensive narrative review. Int J Environ Res Public Health. (2021) 18:5208. doi: 10.3390/ijerph18105208, PMID: 34068866 PMC8153618

[11] Clemente-Suárez VJ Redondo-Flórez L Rubio-Zarapuz A Martínez-Guardado I Navarro-Jiménez E Tornero-Aguilera JF. Nutritional and exercise interventions in Cancer-related Cachexia: An extensive narrative review. Int J Environ Res Public Health. (2022) 19:4604. doi: 10.3390/ijerph19084604, PMID: 35457471 PMC9025820

[12] Clemente-Suárez VJ Navarro-Jiménez E Jimenez M Hormeño-Holgado A Martinez-Gonzalez MB Benitez-Agudelo JC. *Impact of COVID-19 pandemic in public mental health: An Extensive Narrative Review Sustainability* (2021).

[13] Wong GWK Leung TF Ko FWS. Changing prevalence of allergic diseases in the Asia-Pacific region. Allergy Asthma Immunol Res. (2013) 5:251–7. doi: 10.4168/aair.2013.5.5.251, PMID: 24003381 PMC3756171

[14] Podlecka D Gromadzińska J Mikołajewska K Fijałkowska B Stelmach I Jerzynska J. Longitudinal effect of phthalates exposure on allergic diseases in children. Ann Allergy Asthma Immunol. (2020) 125:84–9. doi: 10.1016/j.anai.2020.03.022, PMID: 32244034

[15] To T Zhu J Stieb D Gray N Fong I Pinault L. Early life exposure to air pollution and incidence of childhood Asthma, allergic rhinitis and eczema. Eur Respir J. (2020) 55:1900913. doi: 10.1183/13993003.00913-2019, PMID: 31806712 PMC7031706

[16] Yang C-F Yang C-C Wang I-J. Association between allergic diseases, allergic sensitization and attention-deficit/hyperactivity disorder in children: a large-scale, population-based study. J Chin Med Assoc. (2018) 81:277–83. doi: 10.1016/j.jcma.2017.07.016, PMID: 29239851

[17] Hong S-J Ahn K-M Lee S-Y Kim K-E. The Prevalences of Asthma and allergic diseases in Korean children. Clin Exp Pediatr. (2008) 51:343–50. doi: 10.3346/jkms.2001.16.2.155, PMID: 11306740 PMC3054735

[18] Julia V Macia L Dombrowicz D. The impact of diet on Asthma and allergic diseases. Nat Rev Immunol. (2015) 15:308–22. doi: 10.1038/nri3830, PMID: 25907459

[19] Wardlaw AJ Brightling C Green R Woltmann G Pavord I. Eosinophils in Asthma and other allergic diseases. Br Med Bull. (2000) 56:985–1003. doi: 10.1258/0007142001903490, PMID: 11359633

[20] Wüthrich B Schmid-Grendelmeier P Schindler C Imboden M Bircher A Zemp E. Prevalence of atopy and respiratory allergic diseases in the elderly SAPALDIA population. Int Arch Allergy Immunol. (2013) 162:143–8. doi: 10.1159/000351416, PMID: 23921456

[21] Scordamaglia F Balsamo M Scordamaglia A Moretta A Mingari MC Canonica GW. Perturbations of natural killer cell regulatory functions in respiratory allergic diseases. J Allergy Clin Immunol. (2008) 121:479–85. doi: 10.1016/j.jaci.2007.09.047, PMID: 18061653

[22] D’Amato G Cecchi L. Effects of climate change on environmental factors in respiratory allergic diseases. Clin Exp allergy J Br Soc Allergy Clin Immunol. (2008) 38:1264–74. doi: 10.1111/j.1365-2222.2008.03033.x, PMID: 18537982

[23] Joshi M Goraya H Joshi A Bartter T. Climate change and respiratory diseases: a 2020 perspective. Curr Opin Pulm Med. (2020) 26:119–27. doi: 10.1097/MCP.0000000000000656, PMID: 31851023

[24] D’Amato G Chong-Neto HJ Monge Ortega OP Vitale C Ansotegui I Rosario N. The effects of climate change on respiratory allergy and Asthma induced by pollen and Mold allergens. Allergy. (2020) 75:2219–28. doi: 10.1111/all.14476, PMID: 32589303

[25] Barnes CS. Impact of climate change on pollen and respiratory disease. Curr Allergy Asthma Rep. (2018) 18:1–11. doi: 10.1007/s11882-018-0813-7, PMID: 30238321

[26] Ziska LH Makra L Harry SK Bruffaerts N Hendrickx M Coates F. Temperature-related changes in airborne allergenic pollen abundance and seasonality across the northern hemisphere: a retrospective data analysis. Lancet Planet Heal. (2019) 3:e124–31. doi: 10.1016/S2542-5196(19)30015-4, PMID: 30904111

[27] Cecchi L D’Amato G Ayres JG Galan C Forastiere F Forsberg B. Projections of the effects of climate change on allergic Asthma: the contribution of aerobiology. Allergy. (2010) 65:1073–81. doi: 10.1111/j.1398-9995.2010.02423.x, PMID: 20560904

[28] Dave ND Xiang L Rehm KE Marshall GDJ. Stress and allergic diseases. Immunol Allergy Clin N Am. (2011) 31:55–68. doi: 10.1016/j.iac.2010.09.009, PMID: 21094923 PMC3264048

[29] González-Diaz SN Arias-Cruz A Macouzet-Sánchez C Partida-Ortega AB. Impact of air pollution in respiratory allergic diseases. Med Univ. (2016) 18:212–5. doi: 10.1016/j.rmu.2016.10.006

[30] Nicolussi FH Santos AP André SC Veiga TB Takayanagui AM. Air pollution and respiratory allergic diseases in schoolchildren. Rev Saude Publica. (2014) 48:326–30. doi: 10.1590/s0034-8910.2014048004940, PMID: 24897055 PMC4206145

[31] Jenerowicz D Silny W Dańczak-Pazdrowska A Polańska A Osmola-Mańkowska A Olek-Hrab K. Environmental factors and allergic diseases. Ann Agric Environ Med. (2012) 19:475–81. PMID: 23020042

[32] Branum AM Lukacs SL. Food allergy among children in the United States. Pediatrics. (2009) 124:1549–55. doi: 10.1542/peds.2009-1210, PMID: 19917585

[33] Wüthrich B. Epidemiology of the allergic diseases: are they really on the increase? Int Arch Allergy Appl Immunol. (1989) 90:3–10. doi: 10.1159/000235067, PMID: 2613351

[34] Aberg N Sundell J Eriksson B Hesselmar B Aberg B. Prevalence of allergic diseases in schoolchildren in relation to family history, upper respiratory infections, and residential characteristics. Allergy. (1996) 51:232–7. doi: 10.1111/j.1398-9995.1996.tb04598.x, PMID: 8792919

[35] Traina G Barbalace A Betti F Bolzacchini E Bonini M Contini D. What impact of air pollution in pediatric respiratory allergic diseases. Pediatr. Allergy Immunol. (2020) 31:26–8. doi: 10.1111/pai.13362, PMID: 33236436

[36] Araujo LML Rosario NA Mari A. Molecular-based diagnosis of respiratory allergic diseases in children from Curitiba, a City in southern Brazil. Allergol Immunopathol (Madr). (2016) 44:18–22. doi: 10.1016/j.aller.2015.03.001, PMID: 25982581

[37] Jaber R. Respiratory and allergic diseases: from upper respiratory tract infections to Asthma. Prim Care. (2002) 29:231–61. doi: 10.1016/s0095-4543(01)00008-2, PMID: 12391710

[38] Cogswell JJ Mitchell EB Alexander J. Parental smoking, breast feeding, and respiratory infection in development of allergic diseases. Arch Dis Child. (1987) 62:338–44. doi: 10.1136/adc.62.4.338, PMID: 3592725 PMC1778366

[39] Hong S Son DK Lim WR Kim SH Kim H Yum HY. The prevalence of atopic dermatitis, Asthma, and allergic rhinitis and the comorbidity of allergic diseases in children. Environ Health Toxicol. (2012) 27:e2012006. doi: 10.5620/eht.2012.27.e2012006, PMID: 22359737 PMC3282234

[40] Bunyavanich S Schadt EE. Systems biology of Asthma and allergic diseases: a multiscale approach. J Allergy Clin Immunol. (2015) 135:31–42. doi: 10.1016/j.jaci.2014.10.015, PMID: 25468194 PMC4289105

[41] Breiteneder H Peng Y-Q Agache I Diamant Z Eiwegger T Fokkens WJ. Biomarkers for diagnosis and prediction of therapy responses in allergic diseases and Asthma. Allergy. (2020) 75:3039–68. doi: 10.1111/all.14582, PMID: 32893900 PMC7756301

[42] Moustaki M Loukou I Tsabouri S Douros K. The links between allergen exposure and sensitization in children and adolescents: an overview for the clinician. Expert Rev Clin Immunol. (2022) 18:581–90. PMID: 35502686 10.1080/1744666X.2022.2072297

[43] Davis EC Jackson CM Ting T Harizaj A Järvinen KM. Predictors and biomarkers of food allergy and sensitization in early childhood. Ann Allergy Asthma Immunol. (2022) 129:292–300. doi: 10.1016/j.anai.2022.04.025, PMID: 35490857 PMC11910167

[44] Renz H Skevaki C. Early life microbial exposures and allergy risks: opportunities for prevention. Nat Rev Immunol. (2021) 21:177–91. doi: 10.1038/s41577-020-00420-y, PMID: 32918062

[45] Murrison LB Brandt EB Myers JB Hershey GK. Environmental exposures and mechanisms in allergy and Asthma development. J Clin Invest. (2019) 129:1504–15. doi: 10.1172/JCI124612, PMID: 30741719 PMC6436881

[46] Bousquet J Schünemann HJ Togias A Bachert C Erhola M Hellings PW. Next-generation allergic rhinitis and its impact on Asthma (ARIA) guidelines for allergic rhinitis based on grading of recommendations assessment, development and evaluation (GRADE) and Real-world evidence. J Allergy Clin Immunol. (2020) 145:70–80.e3. doi: 10.1016/j.jaci.2019.06.049, PMID: 31627910

[47] Solé D Rosário Filho NA Sarinho ES Camelo-Nunes IC Barreto BAP Medeiros ML. Prevalence of Asthma and allergic diseases in adolescents: nine-year follow-up study (2003-2012). J Pediatr. (2015) 91:30–5. doi: 10.1016/j.jped.2014.05.002, PMID: 25046259

[48] Pali-Schöll I Namazy J Jensen-Jarolim E. Allergic diseases and Asthma in pregnancy, a secondary publication. World Allergy Organ J. (2017) 10:10. doi: 10.1186/s40413-017-0141-8, PMID: 28286601 PMC5333384

[49] Li Y Rui X Ma B Jiang F Chen J. Early-life environmental factors, IFN-$γ$ methylation patterns, and childhood allergic rhinitis. Int Arch Allergy Immunol. (2019) 178:323–32. doi: 10.1159/000495304, PMID: 30612129

[50] Sbihi H Boutin RCT Cutler C Suen M Finlay BB Turvey SE. Thinking bigger: how early-life environmental exposures shape the gut microbiome and influence the development of Asthma and allergic disease. Allergy. (2019) 74:2103–15. doi: 10.1111/all.13812, PMID: 30964945

[51] Kuo N-C Lin C-H Lin M-C. Prenatal and early life exposure to air pollution and the incidence of Kawasaki disease. Sci Rep. (2022) 12:3415. doi: 10.1038/s41598-022-07081-y, PMID: 35233028 PMC8888747

[52] Calzada D Baos S Cremades-Jimeno L Cárdaba B. Immunological mechanisms in allergic diseases and allergen tolerance: the role of Treg cells. J Immunol Res. (2018) 2018:6012053. doi: 10.1155/2018/6012053, PMID: 30013991 PMC6022267

[53] Purcell K Brady K Chai H Muser J Molk L Gordon N. The effect on Asthma in children of experimental separation from the family. Psychosom Med. (1969) 31:144–64. doi: 10.1097/00006842-196903000-00008, PMID: 5784001

[54] Vahlkvist S Inman MD Pedersen S. Effect of Asthma treatment on fitness, daily activity and body composition in children with Asthma. Allergy. (2010) 65:1464–71. doi: 10.1111/j.1398-9995.2010.02406.x, PMID: 20557298

[55] Orellano P Quaranta N Reynoso J Balbi B Vasquez J. Association of Outdoor air Pollution with the prevalence of Asthma in children of Latin America and the Caribbean: a systematic review and Meta-analysis. J Asthma. (2018) 55:1174–86. doi: 10.1080/02770903.2017.1402342 29211546

[56] Ranjbari M Shams Esfandabadi Z Zanetti MC Scagnelli SD Siebers P-O Aghbashlo M. Three pillars of sustainability in the wake of COVID-19: a systematic review and future research agenda for sustainable development. J Clean Prod. (2021) 297:126660. doi: 10.1016/j.jclepro.2021.126660, PMID: 34785869 PMC8580193

[57] El-Husseini ZW Gosens R Dekker F Koppelman GH. The genetics of Asthma and the promise of genomics-guided drug target discovery. Lancet Respir Med. (2020) 8:1045–56. doi: 10.1016/S2213-2600(20)30363-5, PMID: 32910899

[58] Mallol J Crane J von Mutius E Odhiambo J Keil U Stewart A. The international study of Asthma and allergies in childhood (ISAAC) phase three: a global synthesis. Allergol Immunopathol (Madr). (2013) 41:73–85. doi: 10.1016/j.aller.2012.03.001, PMID: 22771150

[59] Leung ASY Tham EH Li J Pacharn P Takizawa T Lee E. The role of the environment in shaping the trends of childhood Asthma - An Asian perspective. Pediatr Allergy Immunol. (2021) 32:1152–64. doi: 10.1111/pai.13508, PMID: 33760296

[60] Lambrecht BN Hammad H. The immunology of the allergy epidemic and the hygiene hypothesis. Nat Immunol. (2017) 18:1076–83. doi: 10.1038/ni.3829, PMID: 28926539

[61] Ege MJ Mayer M Normand A-C Genuneit J Cookson WOCM Braun-Fahrländer C. Exposure to environmental microorganisms and childhood Asthma. N Engl J Med. (2011) 364:701–9. doi: 10.1056/NEJMoa1007302, PMID: 21345099

[62] Feng M Yang Z Pan L Lai X Xian M Huang X. Associations of early life exposures and environmental factors with Asthma among children in rural and urban areas of Guangdong. China Chest. (2016) 149:1030–41. doi: 10.1016/j.chest.2015.12.028, PMID: 26836923

[63] Stein MM Hrusch CL Gozdz J Igartua C Pivniouk V Murray SE. Innate immunity and Asthma risk in Amish and Hutterite farm children. N Engl J Med. (2016) 375:411–21. doi: 10.1056/NEJMoa1508749, PMID: 27518660 PMC5137793

[64] Riedler J Braun-Fahrländer C Eder W Schreuer M Waser M Maisch S. Exposure to farming in early life and development of Asthma and allergy: a cross-sectional survey. Lancet (London, England). (2001) 358:1129–33. doi: 10.1016/S0140-6736(01)06252-3, PMID: 11597666

[65] Lampi J Koskela H Hartikainen A-L Ramasamy A Couto Alves A Järvelin M-R. Farm environment during infancy and lung function at the age of 31: a prospective birth cohort study in Finland. BMJ Open. (2015) 5:e007350. doi: 10.1136/bmjopen-2014-007350, PMID: 26201721 PMC4513452

[66] Genuneit J Strachan DP Büchele G Weber J Loss G Sozanska B. The combined effects of family size and farm exposure on childhood Hay fever and atopy. Pediatr Allergy Immunol. (2013) 24:293–8. doi: 10.1111/pai.12053, PMID: 23551831

[67] Lee-Sarwar K. The farm-like effect: rural exposures in early life, the microbiome, and Asthma. J Allergy Clin Immunol. (2021):89–90. doi: 10.1016/j.jaci.2021.04.020, PMID: 33915125

[68] Lehtimäki J Thorsen J Rasmussen MA Hjelmsø M Shah S Mortensen MS. Urbanized microbiota in infants, immune constitution, and later risk of atopic diseases. J Allergy Clin Immunol. (2021) 148:234–43. doi: 10.1016/j.jaci.2020.12.621, PMID: 33338536

[69] Cavaleiro Rufo J Paciência I Hoffimann E Moreira A Barros H Ribeiro AI. The Neighbourhood natural environment is associated with Asthma in children: a birth cohort study. Allergy. (2021) 76:348–58. doi: 10.1111/all.14493, PMID: 32654186

[70] Dadvand P Villanueva CM Font-Ribera L Martinez D Basagaña X Belmonte J. Risks and benefits of Green spaces for children: a cross-sectional study of associations with sedentary behavior, obesity, Asthma, and allergy. Environ Health Perspect. (2014) 122:1329–35. doi: 10.1289/ehp.1308038, PMID: 25157960 PMC4256701

[71] Andrusaityte S Grazuleviciene R Kudzyte J Bernotiene A Dedele A Nieuwenhuijsen MJ. Associations between Neighbourhood greenness and Asthma in preschool children in Kaunas, Lithuania: a case-control study. BMJ Open. (2016) 6:e010341. doi: 10.1136/bmjopen-2015-010341 PMC4838715 27067890

[72] Paciência I Rufo JC Silva D Martins C Mendes F Rama T. School environment associates with lung function and autonomic nervous system activity in children: a cross-sectional study. Sci Rep. (2019) 9:15156. doi: 10.1038/s41598-019-51659-y, PMID: 31641175 PMC6805928

[73] Ribeiro AI Tavares C Guttentag A Barros H. Association between Neighbourhood Green space and biological markers in school-aged children. Findings from the generation XXI birth cohort. Environ Int. (2019) 132:105070. doi: 10.1016/j.envint.2019.105070, PMID: 31387021

[74] Ball TM Castro-Rodriguez JA Griffith KA Holberg CJ Martinez FD Wright AL. Siblings, Day-care attendance, and the risk of Asthma and wheezing during childhood. N Engl J Med. (2000) 343:538–43. doi: 10.1056/NEJM200008243430803, PMID: 10954761

[75] Celedón JC Milton DK Ramsey CD Litonjua AA Ryan L Platts-Mills TAE. Exposure to dust mite allergen and endotoxin in early life and Asthma and atopy in childhood. J Allergy Clin Immunol. (2007) 120:144–9. doi: 10.1016/j.jaci.2007.03.037, PMID: 17507083 PMC3737770

[76] Lødrup Carlsen KC Roll S Carlsen K-H Mowinckel P Wijga AH Brunekreef B. Does pet ownership in infancy Lead to Asthma or allergy at school age? Pooled analysis of individual participant data from 11 European birth cohorts. PLoS One. (2012) 7:e43214. doi: 10.1371/journal.pone.0043214, PMID: 22952649 PMC3430634

[77] Fall T Lundholm C Örtqvist AK Fall K Fang F Hedhammar Å. Early exposure to dogs and farm animals and the risk of childhood Asthma. JAMA Pediatr. (2015) 169:e153219. doi: 10.1001/jamapediatrics.2015.3219, PMID: 26523822

[78] Braun-Fahrländer C Riedler J Herz U Eder W Waser M Grize L. Environmental exposure to endotoxin and its relation to Asthma in school-age children. N Engl J Med. (2002) 347:869–77. doi: 10.1056/NEJMoa020057, PMID: 12239255

[79] Giovannangelo M Gehring U Nordling E Oldenwening M Terpstra G Bellander T. Determinants of house dust endotoxin in three European countries - the AIRALLERG study. Indoor Air. (2007) 17:70–9. doi: 10.1111/j.1600-0668.2006.00461.x, PMID: 17257154

[80] Gozdz J Holbreich M Metwali N Thorne PS Sperling AI Martinez FD. Amish and Hutterite environmental farm products have opposite effects on experimental models of Asthma. Ann Am Thorac Soc. (2016) 13:S99. doi: 10.1513/AnnalsATS.201509-581MG, PMID: 27027969 PMC5015741

[81] Depner M Ege MJ Cox MJ Dwyer S Walker AW Birzele LT. Bacterial microbiota of the upper respiratory tract and childhood Asthma. J Allergy Clin Immunol. (2017) 139:826–834.e13. doi: 10.1016/j.jaci.2016.05.050, PMID: 27576124

[82] Peplonska B Safranow K Adamczyk J Boguszewski D Szymański K Soltyszewski I. Association of Serotoninergic Pathway Gene Variants with elite athletic status in the polish population. J Sports Sci. (2019) 37:1655–62. doi: 10.1080/02640414.2019.1583156, PMID: 30836829

[83] Lynch SV Wood RA Boushey H Bacharier LB Bloomberg GR. Effects of early-life exposure to allergens and Bacteria on recurrent wheeze and atopy in urban children. J Allergy Clin Immunol. (2014) 134:593–601.e12. doi: 10.1016/j.jaci.2014.04.018, PMID: 24908147 PMC4151305

[84] Schuijs MJ Willart MA Vergote K Gras D Deswarte K Ege MJ. Farm dust and endotoxin protect against allergy through A20 induction in lung epithelial cells. Science. (2015) 349:1106–10. doi: 10.1126/science.aac6623, PMID: 26339029

[85] Holt PG Sly PD. Environmental microbial exposure and protection against Asthma. N Engl J Med. (2015) 373:2576–8. doi: 10.1056/NEJMcibr1511291, PMID: 26699175

[86] Gosalbes MJ Llop S Vallès Y Moya A Ballester F Francino MP. Meconium microbiota types dominated by lactic acid or enteric Bacteria are differentially associated with maternal eczema and respiratory problems in infants. Clin Exp Allergy. (2013) 43:198–211. doi: 10.1111/cea.12063, PMID: 23331561

[87] Lynch SV Pedersen O. The human intestinal microbiome in health and disease. N Engl J Med. (2016) 375:2369–79. doi: 10.1056/NEJMra1600266, PMID: 27974040

[88] Lynch SV Boushey HA. The microbiome and development of allergic disease. Curr Opin Allergy Clin Immunol. (2016) 16:165–71. doi: 10.1097/ACI.0000000000000255, PMID: 26885707 PMC5378446

[89] Rizzardi KF da Silva Toledo E Ferraz LFC Darrieux M Girardello R de Lima Marson FA. Association between Asthma and enamel defects in primary and Young permanent teeth - a systematic review. Pediatr Pulmonol. (2022) 57:26–37. doi: 10.1002/ppul.25737, PMID: 34698451

[90] Sozańska B. Microbiome in the primary prevention of allergic diseases and bronchial Asthma. Allergol Immunopathol (Madr). (2019) 47:79–84. doi: 10.1016/j.aller.2018.03.005, PMID: 29980403

[91] Herbst T Sichelstiel A Schär C Yadava K Bürki K Cahenzli J. Dysregulation of allergic airway inflammation in the absence of microbial colonization. Am J Respir Crit Care Med. (2011) 184:198–205. doi: 10.1164/rccm.201010-1574OC, PMID: 21471101

[92] Conrad ML Ferstl R Teich R Brand S Blümer N Yildirim AO. Maternal TLR signaling is required for prenatal Asthma protection by the nonpathogenic microbe *Acinetobacter lwoffii* F78. J Exp Med. (2009) 206:2869–77. doi: 10.1084/jem.20090845, PMID: 19995952 PMC2806458

[93] Loss G Bitter S Wohlgensinger J Frei R Roduit C Genuneit J. Prenatal and early-life exposures Alter expression of innate immunity genes: the PASTURE cohort study. J Allergy Clin Immunol. (2012) 130:523–30.e9. doi: 10.1016/j.jaci.2012.05.049, PMID: 22846753

[94] Teo SM Mok D Pham K Kusel M Serralha M Troy N. The infant nasopharyngeal microbiome impacts severity of lower respiratory infection and risk of Asthma development. Cell Host Microbe. (2015) 17:704–15. doi: 10.1016/j.chom.2015.03.008, PMID: 25865368 PMC4433433

[95] Stokholm J Blaser MJ Thorsen J Rasmussen MA Waage J Vinding RK. Maturation of the gut microbiome and risk of Asthma in childhood. Nat Commun. (2018) 9:141. doi: 10.1038/s41467-017-02573-2, PMID: 29321519 PMC5762761

[96] McEwen BS. The neurobiology of stress: from serendipity to clinical relevance. Brain Res. (2000) 886:–189. doi: 10.1016/s0006-8993(00)02950-4, PMID: 11119695

[97] Pace TWW Negi LT Adame DD Cole SP Sivilli TI Brown TD. Effect of compassion meditation on neuroendocrine, innate immune and behavioral responses to psychosocial stress. Psychoneuroendocrinology. (2009) 34:87–98. doi: 10.1016/j.psyneuen.2008.08.011, PMID: 18835662 PMC2695992

[98] Eisenberger NI Cole SW. Social neuroscience and health: neurophysiological mechanisms linking social ties with physical health. Nat Neurosci. (2012) 15:669–74. doi: 10.1038/nn.3086, PMID: 22504347

[99] Barnes PJ. Cellular and molecular mechanisms of Asthma and COPD. Clin Sci (Lond). (2017) 131:1541–58. doi: 10.1042/CS20160487, PMID: 28659395

[100] Wenzel SE. Emergence of biomolecular pathways to define novel Asthma phenotypes. Type-2 immunity and beyond. Am J Respir Cell Mol Biol. (2016):1–4. doi: 10.1165/rcmb.2016-0141PS, PMID: 27164162 PMC4942211

[101] Dimitrova D Youroukova V Ivanova-Todorova E Tumangelova-Yuzeir K Velikova T. Serum levels of IL-5, IL-6, IL-8, IL-13 and IL-17A in pre-defined groups of adult patients with moderate and severe bronchial Asthma. Respir Med. (2019) 154:144–54. doi: 10.1016/j.rmed.2019.06.024, PMID: 31260861

[102] Ghosh S Hayden MS. Celebrating 25 years of NF-ΚB research. Immunol Rev. (2012):5–13. doi: 10.1111/j.1600-065X.2012.01111.x, PMID: 22435544 PMC3313446

[103] Mishra V Banga J Silveyra P. Oxidative stress and cellular pathways of Asthma and inflammation: therapeutic strategies and pharmacological targets. Pharmacol Ther. (2018) 181:169–82. doi: 10.1016/j.pharmthera.2017.08.011, PMID: 28842273 PMC5743757

[104] Reijmerink NE Bottema RWB Kerkhof M Gerritsen J Stelma FF Thijs C. TLR-related pathway analysis: novel gene-gene interactions in the development of Asthma and atopy. Allergy. (2010) 65:199–207. doi: 10.1111/j.1398-9995.2009.02111.x, PMID: 19968634

[105] Tesse R Pandey RC Kabesch M. Genetic variations in toll-like receptor pathway genes influence Asthma and atopy. Allergy. (2011) 66:307–16. doi: 10.1111/j.1398-9995.2010.02489.x, PMID: 21039600

[106] Vercelli D. Discovering susceptibility genes for Asthma and allergy. Nat Rev Immunol. (2008) 8:169–82. doi: 10.1038/nri2257, PMID: 18301422

[107] Brasier AR. The NF-KappaB regulatory network. Cardiovasc Toxicol. (2006) 6:111–30. doi: 10.1385/ct:6:2:111, PMID: 17303919

[108] Kim DH Gu A Lee J-S Yang EJ Kashif A Hong MH. Suppressive effects of S100A8 and S100A9 on neutrophil apoptosis by cytokine release of human bronchial epithelial cells in Asthma. Int J Med Sci. (2020) 17:498–509. doi: 10.7150/ijms.37833, PMID: 32174780 PMC7053304

[109] Rahal A Kumar A Singh V Yadav B Tiwari R Chakraborty S. Oxidative stress, Prooxidants, and antioxidants: the interplay. Biomed Res Int. (2014) 2014:761264. doi: 10.1155/2014/761264, PMID: 24587990 PMC3920909

[110] Suzuki S Matsukura S Takeuchi H Kawaguchi M Ieki K Odaka M. Increase in reactive oxygen metabolite level in acute exacerbations of Asthma. Int Arch Allergy Immunol. (2008) 146:67–72. doi: 10.1159/000126064, PMID: 18504410

[111] Al-Harbi NO Nadeem A Al-Harbi MM Imam F Al-Shabanah OA Ahmad SF. Oxidative airway inflammation leads to systemic and vascular oxidative stress in a murine model of allergic Asthma. Int Immunopharmacol. (2015) 26:237–45. doi: 10.1016/j.intimp.2015.03.032, PMID: 25843257

[112] Erzurum SC. New insights in oxidant biology in Asthma. Ann Am Thorac Soc. (2016) 13:S35–9. doi: 10.1513/AnnalsATS.201506-385MG, PMID: 27027950 PMC5015728

[113] Uchida M Anderson EL Squillace DL Patil N Maniak PJ Iijima K. Oxidative stress serves as a key checkpoint for IL-33 release by airway epithelium. Allergy. (2017) 72:1521–31. doi: 10.1111/all.13158, PMID: 28273344 PMC5591045

[114] Davoine F Lacy P. Eosinophil cytokines, chemokines, and growth factors: emerging roles in immunity. Front Immunol. (2014) 5:570. doi: 10.3389/fimmu.2014.00570, PMID: 25426119 PMC4225839

[115] Lee JJ Jacobsen EA Ochkur SI McGarry MP Condjella RM Doyle AD. Human versus mouse eosinophils: “that which we call an eosinophil, by any other name would stain as red”. J Allergy Clin Immunol. (2012) 130:572–84. doi: 10.1016/j.jaci.2012.07.025, PMID: 22935586 PMC3496419

[116] Piliponsky AM Pickholtz D Gleich GJ Levi-Schaffer F. Human eosinophils induce histamine release from antigen-activated rat peritoneal mast cells: a possible role for mast cells in late-phase allergic reactions. J Allergy Clin Immunol. (2001) 107:993–1000. doi: 10.1067/mai.2001.114656, PMID: 11398076

[117] Wynn TA. Type 2 cytokines: mechanisms and therapeutic strategies. Nat Rev Immunol. (2015) 15:271–82. doi: 10.1038/nri3831 25882242

[118] Fulkerson PC Schollaert KL Bouffi C Rothenberg ME. IL-5 triggers a cooperative cytokine network that promotes eosinophil precursor maturation. J Immunol. (2014) 193:4043–52. doi: 10.4049/jimmunol.1400732, PMID: 25230753 PMC4185228

[119] Bisgaard H. Pathophysiology of the Cysteinyl leukotrienes and effects of leukotriene receptor antagonists in Asthma. Allergy. (2001) 56:7–11. doi: 10.1034/j.1398-9995.56.s66.2.x, PMID: 11421935

[120] Halwani R Al-Muhsen S Hamid Q. Airway remodeling in Asthma. Curr Opin Pharmacol. (2010) 10:236–45. doi: 10.1016/j.coph.2010.06.004, PMID: 20591736

[121] Halwani R Al-Muhsen S Al-Jahdali H Hamid Q. Role of transforming growth factor-β in airway remodeling in Asthma. Am J Respir Cell Mol Biol. (2011) 44:127–33. doi: 10.1165/rcmb.2010-0027TR, PMID: 20525803

[122] Potaczek DP Miethe S Schindler V Alhamdan F Garn H. Role of airway epithelial cells in the development of different Asthma phenotypes. Cell Signal. (2020) 69:109523. doi: 10.1016/j.cellsig.2019.109523, PMID: 31904412

[123] O’Flaherty SM Sutummaporn K Häggtoft WL Worrall AP Rizzo M Braniste V. TLR-stimulated eosinophils mediate recruitment and activation of NK cells in vivo. Scand J Immunol. (2017) 85:417–24. doi: 10.1111/sji.12554, PMID: 28426135

[124] Van Hulst G Batugedara HM Jorssen J Louis R Bureau F Desmet CJ. Eosinophil diversity in Asthma. Biochem Pharmacol. (2020) 179:113963. doi: 10.1016/j.bcp.2020.113963, PMID: 32278006

[125] Niraula A Witcher KG Sheridan JF Godbout JP. Interleukin-6 induced by social stress promotes a unique transcriptional signature in the monocytes that facilitate anxiety. Biol Psychiatry. (2019) 85:679–89. doi: 10.1016/j.biopsych.2018.09.030, PMID: 30447911 PMC6440848

[126] Miyasaka T Dobashi-Okuyama K Takahashi T Takayanagi M Ohno I. The interplay between neuroendocrine activity and psychological stress-induced exacerbation of allergic Asthma. Allergol Int Off J Japanese Soc Allergol. (2018) 67:32–42. doi: 10.1016/j.alit.2017.04.013, PMID: 28539203

[127] Zammit C Liddicoat H Moonsie I Makker H. Obesity and respiratory diseases. Int J Gen Med. (2010) 3:335–43. doi: 10.2147/IJGM.S11926, PMID: 21116339 PMC2990395

[128] Su X Ren Y Li M Zhao X Kong L Kang J. Prevalence of comorbidities in Asthma and nonasthma patients: a Meta-analysis. Medicine (Baltimore). (2016) 95:e3459. doi: 10.1097/MD.0000000000003459, PMID: 27258489 PMC4900697

[129] Adeyeye OO Ogbera AO Ogunleye OO Brodie-Mens AT Abolarinwa FF Bamisile RT. Understanding Asthma and the metabolic syndrome - a Nigerian report. Int Arch Med. (2012) 5:20. doi: 10.1186/1755-7682-5-20, PMID: 22726248 PMC3499319

[130] Mueller NT Koh W-P Odegaard AO Gross MD Yuan J-M Pereira MA. Asthma and the risk of type 2 diabetes in the Singapore Chinese health study. Diabetes Res Clin Pract. (2013) 99:192–9. doi: 10.1016/j.diabres.2012.11.019, PMID: 23260853 PMC3615124

[131] Song Y Klevak A Manson JE Buring JE Liu S. Asthma, chronic obstructive pulmonary disease, and type 2 diabetes in the Women’s health study. Diabetes Res Clin Pract. (2010) 90:365–71. doi: 10.1016/j.diabres.2010.09.010, PMID: 20926152 PMC2993844

[132] Brumpton BM Camargo CAJ Romundstad PR Langhammer A Chen Y Mai X-M. Metabolic syndrome and incidence of Asthma in adults: the HUNT study. Eur Respir J. (2013) 42:1495–502. doi: 10.1183/09031936.00046013, PMID: 23845717

[133] Lee KH Lee HS. Hypertension and diabetes mellitus as risk factors for Asthma in Korean adults: the sixth Korea National Health and nutrition examination survey. Int Health. (2020) 12:246–52. doi: 10.1093/inthealth/ihz067, PMID: 31608936 PMC7322203

[134] Verbovoy AF Kosareva OV Akhmerova RI. Leptin, resistin, and hormonal and metabolic parameters in women with type 2 diabetes and in those with its concurrence with asthma. Ter Arkh. (2015) 87:37–41. doi: 10.17116/terarkh2015871037-41, PMID: 26978172

[135] Rayner L McGovern A Creagh-Brown B Woodmansey C de Lusignan S. Type 2 diabetes and Asthma: systematic review of the bidirectional relationship. Curr Diabetes Rev. (2019) 15:118–26. doi: 10.2174/1573399814666180711114859, PMID: 29992891

[136] Gershon AS Guan J Wang C Victor JC To, T. Describing and quantifying Asthma comorbidity [corrected]: a population study. PLoS One. (2012) 7:e34967. doi: 10.1371/journal.pone.0034967, PMID: 22586445 PMC3346768

[137] Yun HD Knoebel E Fenta Y Gabriel SE Leibson CL Loftus EVJ. Asthma and Proinflammatory conditions: a population-based retrospective matched cohort study. Mayo Clin Proc. (2012) 87:953–60. doi: 10.1016/j.mayocp.2012.05.020, PMID: 22980164 PMC3538394

[138] Wytrychowski K Obojski A Hans-Wytrychowska A. The influence of insulin therapy on the course of acute exacerbation of bronchial Asthma. Adv Exp Med Biol. (2016) 884:45–51. doi: 10.1007/5584_2015_175, PMID: 26453066

[139] Klein OL Aviles-Santa L Cai J Collard HR Kanaya AM Kaplan RC. Hispanics/Latinos with type 2 diabetes have functional and symptomatic pulmonary impairment mirroring kidney Microangiopathy: findings from the Hispanic community health study/study of Latinos (HCHS/SOL). Diabetes Care. (2016) 39:2051–7. doi: 10.2337/dc16-1170, PMID: 27612502 PMC5079610

[140] Koskela HO Salonen PH Romppanen J Niskanen L. A history of diabetes but not Hyperglycaemia during exacerbation of obstructive lung disease has impact on long-term mortality: a prospective, observational cohort study. BMJ Open. (2015) 5:e006794. doi: 10.1136/bmjopen-2014-006794, PMID: 25633287 PMC4316436

[141] Kankaanranta H Kauppi P Tuomisto LE Ilmarinen P. Emerging comorbidities in adult Asthma: risks, clinical associations, and mechanisms. Mediat Inflamm. (2016) 2016:3690628. doi: 10.1155/2016/3690628, PMID: 27212806 PMC4861800

[142] Wee JH Park MW Min C Byun SH Park B Choi HG. Association between Asthma and cardiovascular disease. Eur J Clin Investig. (2021) 51:e13396. doi: 10.1111/eci.13396, PMID: 32888313

[143] Wong BW Meredith A Lin D McManus BM. The biological role of inflammation in atherosclerosis. Can J Cardiol. (2012) 28:631–41. doi: 10.1016/j.cjca.2012.06.023, PMID: 22985787

[144] Carpaij OA van den Berge M. The Asthma-obesity relationship: underlying mechanisms and treatment implications. Curr Opin Pulm Med. (2018) 24:42–9. doi: 10.1097/MCP.0000000000000446, PMID: 29176481

[145] Torres RM Souza MDS Coelho ACC de Mello LM Souza-Machado C. Association between Asthma and type 2 diabetes mellitus: mechanisms and impact on Asthma control-a literature review. Can Respir J. (2021) 2021:8830439. doi: 10.1155/2021/8830439, PMID: 33520042 PMC7817304

[146] Scichilone N Rizzo M Benfante A Catania R Giglio RV Nikolic D. Serum low density lipoprotein subclasses in Asthma. Respir Med. (2013) 107:1866–72. doi: 10.1016/j.rmed.2013.09.001, PMID: 24075885

[147] Rasmussen F Hancox RJ Nair P Hansen HS Siersted HC Nybo M. Associations between airway Hyperresponsiveness, obesity and lipoproteins in a longitudinal cohort. Clin Respir J. (2013) 7:268–75. doi: 10.1111/crj.12000, PMID: 22906044

[148] Serafino-Agrusa L Spatafora M Scichilone N. Asthma and metabolic syndrome: current knowledge and future perspectives. World J Clin Cases. (2015) 3:285–92. doi: 10.12998/wjcc.v3.i3.285, PMID: 25789301 PMC4360500

[149] Rastogi D Fraser S Oh J Huber AM Schulman Y Bhagtani RH. Inflammation, metabolic dysregulation, and pulmonary function among obese urban adolescents with Asthma. Am J Respir Crit Care Med. (2015) 191:149–60. doi: 10.1164/rccm.201409-1587OC, PMID: 25457349 PMC4347436

[150] Tanaka T Narazaki M Kishimoto T. IL-6 in inflammation, immunity, and disease. Cold Spring Harb Perspect Biol. (2014) 6:a016295. doi: 10.1101/cshperspect.a016295, PMID: 25190079 PMC4176007

[151] Sideleva O Suratt BT Black KE Tharp WG Pratley RE Forgione P. Obesity and Asthma: An inflammatory disease of adipose tissue not the airway. Am J Respir Crit Care Med. (2012) 186:598–605. doi: 10.1164/rccm.201203-0573OC, PMID: 22837379 PMC3480522

[152] Han Y-Y Forno E Celedón JC. Vitamin D insufficiency and Asthma in a US Nationwide study. J Allergy Clin Immunol Pract. (2017) 5:790–796.e1. doi: 10.1016/j.jaip.2016.10.013, PMID: 27913247 PMC5423854

[153] Babar MZM Hussain M Majeed SA. Vitamin D supplementation improves FEV1 in patients of bronchial Asthma. Pakistan J Med Sci. (2017) 33:1144–7. doi: 10.12669/pjms.335.12990, PMID: 29142554 PMC5673723

[154] Kang Q Zhang X Liu S Huang F. Correlation between the vitamin D levels and Asthma attacks in children: evaluation of the effects of combination therapy of atomization inhalation of budesonide, albuterol and vitamin D supplementation on asthmatic patients. Exp Ther Med. (2018) 15:727–32. doi: 10.3892/etm.2017.5436, PMID: 29399078 PMC5772657

[155] Pfeffer PE. Targeting the Exposome: does correcting vitamin D deficiency have potential to treat and prevent Asthma? Expert review of clinical immunology. England April. (2018) 14:241–3. doi: 10.1080/1744666X.2018.1440207 29431528

[156] Ali NS Nanji K. A review on the role of vitamin D in Asthma. Cureus. (2017) 9:e1288. doi: 10.7759/cureus.1288, PMID: 28680776 PMC5491340

[157] Wang M Liu M Wang C Xiao Y An T Zou M. Association between vitamin D status and Asthma control: a Meta-analysis of randomized trials. Respir Med. (2019) 150:85–94. doi: 10.1016/j.rmed.2019.02.016, PMID: 30961957

[158] Berry MA Hargadon B Shelley M Parker D Shaw DE Green RH. Evidence of a role of tumor necrosis factor alpha in refractory Asthma. N Engl J Med. (2006) 354:697–708. doi: 10.1056/NEJMoa050580, PMID: 16481637

[159] Mora JR Iwata M von Andrian UH. Vitamin effects on the immune system: vitamins a and D take Centre stage. Nat Rev Immunol. (2008) 8:685–98. doi: 10.1038/nri2378, PMID: 19172691 PMC2906676

[160] Mann EH Chambers ES Pfeffer PE Hawrylowicz CM. Immunoregulatory mechanisms of vitamin D relevant to respiratory health and Asthma. Ann N Y Acad Sci. (2014) 1317:57–69. doi: 10.1111/nyas.12410, PMID: 24738964

[161] Korn S Hübner M Jung M Blettner M Buhl R. Severe and uncontrolled adult Asthma is associated with vitamin D insufficiency and deficiency. Respir Res. (2013) 14:25. doi: 10.1186/1465-9921-14-25, PMID: 23432854 PMC3648461

[162] Cassim R Russell MA Lodge CJ Lowe AJ Koplin JJ Dharmage SC. The role of circulating 25 Hydroxyvitamin D in Asthma: a systematic review. Allergy. (2015) 70:339–54. doi: 10.1111/all.12583, PMID: 25631639

[163] Huang S-C Gau S-Y Huang J-Y Wu W-J Wei JC-C. Increased risk of hypothyroidism in people with Asthma: evidence from a Real-world population-based study. J Clin Med. (2022) 11:2776. doi: 10.3390/jcm11102776, PMID: 35628903 PMC9146804

[164] Qin Q Liu P Liu L Wang R Yan N Yang J. The increased but non-predominant expression of Th17-and Th1-specific cytokines in Hashimoto’s thyroiditis but not in graves’ disease. Braz J Med Biol Res. (2012) 45:1202–8. doi: 10.1590/s0100-879x2012007500168, PMID: 23090124 PMC3854214

[165] Janyga S Marek B Kajdaniuk D Ogrodowczyk-Bobik M Urbanek A Bułdak Ł. CD4+ cells in autoimmune thyroid disease. Endokrynol Pol. (2021) 72:572–83. doi: 10.5603/EP.a2021.0076, PMID: 34647609

[166] Murphy RC Pavord ID Alam R Altman MC. Management strategies to reduce exacerbations in non-T2 Asthma. J Allergy Clin Immunol Pract. (2021) 9:2588–97. doi: 10.1016/j.jaip.2021.04.033, PMID: 34246435

[167] Bastard J-P Maachi M Lagathu C Kim MJ Caron M Vidal H. Recent advances in the relationship between obesity, inflammation, and insulin resistance. Eur Cytokine Netw. (2006) 17:4–12. PMID: 16613757

[168] Holloway JW Yang IA Ye S. Variation in the toll-like receptor 4 gene and Susceptibility to myocardial infarction. Pharmacogenet Genomics. (2005) 15:15–21. doi: 10.1097/01213011-200501000-00003, PMID: 15864121

[169] Papi A Brightling C Pedersen SE Reddel HK. Asthma. Lancet (London, England). (2018) 391:783–800. doi: 10.1016/S0140-6736(17)33311-1, PMID: 29273246

[170] Steffes MW Gross MD Lee D-H Schreiner PJ Jacobs DRJ. Adiponectin, visceral fat, oxidative stress, and early macrovascular disease: the coronary artery risk development in Young adults study. Obesity (Silver Spring). (2006) 14:319–26. doi: 10.1038/oby.2006.41, PMID: 16571859

[171] Dut R Dizdar EA Birben E Sackesen C Soyer OU Besler T. Oxidative stress and its determinants in the Airways of Children with Asthma. Allergy. (2008) 63:1605–9. doi: 10.1111/j.1398-9995.2008.01766.x, PMID: 19032232

[172] Lugogo NL Bappanad D Kraft M. Obesity, metabolic dysregulation and oxidative stress in Asthma. Biochim Biophys Acta. (2011) 1810:1120–6. doi: 10.1016/j.bbagen.2011.09.004, PMID: 21944975 PMC3786599

[173] Canöz M Erdenen F Uzun H Müderrisoglu C Aydin S. The relationship of inflammatory cytokines with Asthma and obesity. Clin Invest Med. (2008) 31:E373–9. doi: 10.25011/cim.v31i6.4924, PMID: 19032908

[174] Mehta A Sequeira PS Sahoo RC Kaur G. Is bronchial Asthma a risk factor for gingival diseases? A control study. N Y State Dent J. (2009) 75:44–6. PMID: 19280828

[175] Eloot AK Vanobbergen JN De Baets F Martens LC. Oral health and habits in children with Asthma related to severity and duration of condition. Eur J Paediatr Dent. (2004) 5:210–5. PMID: 15606319

[176] Shulman JD Nunn ME Taylor SE Rivera-Hidalgo F. The prevalence of periodontal-related changes in adolescents with Asthma: results of the third annual National Health and nutrition examination survey. Pediatr Dent. (2003) 25:279–84. PMID: 12889707

[177] Thomas MS Parolia A Kundabala M Vikram M. Asthma and Oral health: a review. Aust Dent J. (2010) 55:128–33. doi: 10.1111/j.1834-7819.2010.01226.x, PMID: 20604752

[178] Gani F Caminati M Bellavia F Baroso A Faccioni P Pancera P. Oral health in asthmatic patients: a review: Asthma and its therapy may impact on Oral health. Clin Mol Allergy. (2020) 18:22. doi: 10.1186/s12948-020-00137-2, PMID: 33292326 PMC7648282

[179] Wootton SH Aguilera E Salazar L Hemmert AC Hasbun R. Enhancing pathogen identification in patients with meningitis and a negative gram stain using the BioFire FilmArray®meningitis/encephalitis panel. Ann Clin Microbiol Antimicrob. (2016) 15:1–4. doi: 10.1186/s12941-016-0137-1, PMID: 27101869 PMC4839114

[180] Yalçın SS Emiralioğlu N Yalçın S. Evaluation of blood and tooth element status in Asthma cases: a preliminary case-control study. BMC Pulm Med. (2021) 21:201. doi: 10.1186/s12890-021-01565-9, PMID: 34130672 PMC8204585

[181] Arafa A Aldahlawi S Fathi A. Assessment of the Oral health status of asthmatic children. Eur J Dent. (2017) 11:357–63. doi: 10.4103/ejd.ejd_65_17, PMID: 28932147 PMC5594966

[182] Acharya S Bhat PV Acharya S. Factors affecting Oral health-related quality of life among pregnant women. Int J Dent Hyg. (2009) 7:102–7. doi: 10.1111/j.1601-5037.2008.00351.x, PMID: 19416092

[183] Dahl R. Systemic side effects of inhaled corticosteroids in patients with Asthma. Respir Med. (2006) 100:1307–17. doi: 10.1016/j.rmed.2005.11.020, PMID: 16412623

[184] Chumpitaz-Cerrate V Bellido-Meza JA Chávez-Rimache L Rodríguez-Vargas C. Impact of inhaler use on dental caries in Asthma pediatrics patients: a case-control study. Arch Argent Pediatr. (2020) 118:38–46. doi: 10.5546/aap.2020.eng.38, PMID: 31984694

[185] Wee JH Park MW Min C Park I-S Park B Choi HG. Poor Oral health is associated with Asthma, allergic rhinitis, and atopic dermatitis in Korean adolescents: a cross-sectional study. Medicine (Baltimore). (2020) 99:e21534. doi: 10.1097/MD.0000000000021534, PMID: 32756203 PMC7402906

[186] Tanaka LS Dezan CC Fernandes KBP Ferreira FBDA Walter LRDF Cerci Neto A. The influence of Asthma onset and severity on malocclusion prevalence in children and adolescents. Dental press. J Orthod. (2012) 17:50–7. doi: 10.1590/S2176-94512012000100007

[187] Fabbrizzi A Amedei A Lavorini F Renda T Fontana G. The lung microbiome: clinical and therapeutic implications. Intern Emerg Med. (2019) 14:1241–50. doi: 10.1007/s11739-019-02208-y, PMID: 31667699

[188] Kasubuchi M Hasegawa S Hiramatsu T Ichimura A Kimura I. Dietary gut microbial metabolites, short-chain fatty acids, and host metabolic regulation. Nutrients. (2015) 7:2839–49. doi: 10.3390/nu7042839, PMID: 25875123 PMC4425176

[189] Durack J Huang YJ Nariya S Christian LS Ansel KM Beigelman A. Bacterial biogeography of adult Airways in Atopic Asthma. Microbiome. (2018) 6:104. doi: 10.1186/s40168-018-0487-3, PMID: 29885665 PMC5994066

[190] Durack J Lynch SV Nariya S Bhakta NR Beigelman A Castro M. Features of the bronchial bacterial microbiome associated with atopy, Asthma, and responsiveness to inhaled corticosteroid treatment. J Allergy Clin Immunol. (2017) 140:63–75. doi: 10.1016/j.jaci.2016.08.055, PMID: 27838347 PMC5502827

[191] Denner DR Sangwan N Becker JB Hogarth DK Oldham J Castillo J. Corticosteroid therapy and airflow obstruction influence the bronchial microbiome, which is distinct from that of Bronchoalveolar lavage in asthmatic airways. J Allergy Clin Immunol. (2016) 137:1398–1405.e3. doi: 10.1016/j.jaci.2015.10.017, PMID: 26627545 PMC4860110

[192] Goleva E Jackson LP Harris JK Robertson CE Sutherland ER Hall CF. The effects of airway microbiome on corticosteroid responsiveness in Asthma. Am J Respir Crit Care Med. (2013) 188:1193–201. doi: 10.1164/rccm.201304-0775OC, PMID: 24024497 PMC3863730

[193] Hilty M Burke C Pedro H Cardenas P Bush A Bossley C. Disordered microbial communities in asthmatic airways. PLoS One. (2010) 5:e8578. doi: 10.1371/journal.pone.0008578, PMID: 20052417 PMC2798952

[194] Huang YJ Nariya S Harris JM Lynch SV Choy DF Arron JR. The airway microbiome in patients with severe Asthma: associations with disease features and severity. J Allergy Clin Immunol. (2015) 136:874–84. doi: 10.1016/j.jaci.2015.05.044, PMID: 26220531 PMC4600429

[195] Huang YJ Nelson CE Brodie EL Desantis TZ Baek MS Liu J. Airway microbiota and bronchial Hyperresponsiveness in patients with suboptimally controlled Asthma. J Allergy Clin Immunol. (2011) 127:372–3. doi: 10.1016/j.jaci.2010.10.048, PMID: 21194740 PMC3037020

[196] Kloepfer KM Lee WM Pappas TE Kang TJ Vrtis RF Evans MD. Detection of pathogenic Bacteria during rhinovirus infection is associated with increased respiratory symptoms and Asthma exacerbations. J Allergy Clin Immunol. (2014) 133:1301–7, 1307.e1-3. doi: 10.1016/j.jaci.2014.02.030, PMID: 24698319 PMC4047978

[197] Busse WW Lemanske RFJ Gern JE. Role of viral respiratory infections in Asthma and Asthma exacerbations. Lancet (London, England). (2010) 376:826–34. doi: 10.1016/S0140-6736(10)61380-3, PMID: 20816549 PMC2972660

[198] Fukushima C Matsuse H Tomari S Obase Y Miyazaki Y Shimoda T. Oral candidiasis associated with inhaled corticosteroid use: comparison of fluticasone and beclomethasone. Ann Allergy Asthma Immunol. (2003) 90:646–51. doi: 10.1016/S1081-1206(10)61870-4, PMID: 12839324

[199] Kurt E Yildirim H Kiraz N Orman A Metintas M Akgun Y. Oropharyngeal candidiasis with dry-powdered fluticasone propionate: 500 microg/Day versus 200 microg/Day. Allergol Immunopathol (Madr). (2008) 36:17–20. doi: 10.1157/13115666, PMID: 18261428

[200] Alka K Amberkar VS Mohan Kumar KP Nandini DB Vidyasagar B. Estimation of salivary *Candida albicans* counts in asthmatic adult patients taking anti-asthmatic medication for 3-5 years. J Oral Maxillofac Pathol. (2018) 22:341–6. doi: 10.4103/jomfp.JOMFP_36_17, PMID: 30651678 PMC6306586

[201] Erdougan T Karakaya G Kalyoncu AF. The frequency and risk factors for oropharyngeal candidiasis in adult Asthma patients using inhaled corticosteroids. Turk Thorac J. (2019) 20:136:139. doi: 10.5152/TurkThoracJ.2019.17011916, PMID: 30958987 PMC6453638

[202] Pratesi A Tarantini F Di Bari M. Skeletal muscle: an endocrine organ. Clin Cases Miner Bone Metab. (2013) 10:11–4. doi: 10.11138/ccmbm/2013.10.1.011, PMID: 23858303 PMC3710002

[203] Clemente-Suárez VJ Beltrán-Velasco AI Ramos-Campo DJ Mielgo-Ayuso J Nikolaidis PA Belando N. Physical activity and COVID-19. The basis for an efficient intervention in times of COVID-19 pandemic. Physiol Behav. (2022) 244:113667. doi: 10.1016/j.physbeh.2021.113667 34861297 PMC8632361

[204] Rogeri PS Gasparini SO Martins GL Costa LKF Araujo CC Lugaresi R. Crosstalk between skeletal muscle and immune system: which roles do IL-6 and glutamine play? Front Physiol. (2020) 11:582258. doi: 10.3389/fphys.2020.582258, PMID: 33178046 PMC7596683

[205] Avallone KM McLeish AC. Asthma and aerobic exercise: a review of the empirical literature. J Asthma. (2013) 50:109–16. doi: 10.3109/02770903.2012.759963, PMID: 23252987

[206] Yawn BP Rank MA Bertram SL Wollan PC. Obesity, low levels of physical activity and smoking present opportunities for primary care Asthma interventions: An analysis of baseline data from the Asthma tools study. NPJ Prim care Respir Med. (2015) 25:15058. doi: 10.1038/npjpcrm.2015.58, PMID: 26426429 PMC4590305

[207] Strine TW Mokdad AH Dube SR Balluz LS Gonzalez O Berry JT. The Association of Depression and Anxiety with obesity and unhealthy behaviors among community-dwelling US adults. Gen Hosp Psychiatry. (2008) 30:127–37. doi: 10.1016/j.genhosppsych.2007.12.008, PMID: 18291294

[208] Ford ES Mannino DM Redd SC Moriarty DG Mokdad AH. Determinants of quality of life among people with Asthma: findings from the behavioral risk factor surveillance system. J Asthma. (2004) 41:327–36. doi: 10.1081/jas-120026090, PMID: 15260466

[209] Dogra S Kuk JL Baker J Jamnik V. Exercise is associated with improved Asthma control in adults. Eur Respir J. (2011) 37:318–23. doi: 10.1183/09031936.00182209, PMID: 20530042

[210] Strine TW Balluz LS Ford ES. The associations between smoking, physical inactivity, obesity, and Asthma severity in the general US population. J Asthma. (2007) 44:651–8. doi: 10.1080/02770900701554896, PMID: 17943577

[211] Kuder MM Clark M Cooley C Prieto-Centurion V Danley A Riley I. A systematic review of the effect of physical activity on Asthma outcomes. J Allergy Clin Immunol Pract. (2021). doi: 10.1016/j.jaip.2021.04.048, PMID: 33964510 PMC8434961

[212] Mensah GA Kiley JP Gibbons GH. Generating evidence to inform an update of Asthma clinical practice guidelines: perspectives from the National Heart, Lung, and Blood Institute. J Allergy Clin Immunol. (2018) 142:744–8. doi: 10.1016/j.jaci.2018.07.004, PMID: 30036600

[213] Piercy KL Troiano RP Ballard RM Carlson SA Fulton JE Galuska DA. The physical activity guidelines for Americans. JAMA. (2018) 320:2020–8. doi: 10.1001/jama.2018.14854, PMID: 30418471 PMC9582631

[214] Ford ES Heath GW Mannino DM Redd SC. Leisure-time physical activity patterns among US adults with Asthma. Chest. (2003). doi: 10.1378/chest.124.2.432, PMID: 12907526

[215] Mancuso CA Sayles W Robbins L Phillips EG Ravenell K Duffy C. Barriers and facilitators to healthy physical activity in Asthma patients. J Asthma. (2006). doi: 10.1080/02770900500498584, PMID: 16517430

[216] Bardagi S Agudo A Gonzalez CA Romero PV. Prevalence of exercise-induced airway narrowing in schoolchildren from a Mediterranean town. Am Rev Respir Dis. (1993). doi: 10.1164/ajrccm/147.5.1112, PMID: 8484618

[217] Karjalainen J. Exercise response in 404 Young men with Asthma: no evidence for a late asthmatic reaction. Thorax. (1991) 46:100–4. doi: 10.1136/thx.46.2.100, PMID: 2014489 PMC462958

[218] Storms WW. Asthma associated with exercise. Immunol Allergy Clin N Am. (2005) 25:31–43. doi: 10.1016/j.iac.2004.09.007, PMID: 15579363

[219] Carson KV Chandratilleke MG Picot J Brinn MP Esterman AJ Smith BJ. Physical training for Asthma. Cochrane Database Syst Rev. (2013):CD001116. doi: 10.1002/14651858.CD001116.pub4, PMID: 24085631 PMC11930393

[220] Pakhale S Luks V Burkett A Turner L. Effect of physical training on airway inflammation in bronchial Asthma: a systematic review. BMC Pulm Med. (2013) 13, 38. doi: 10.1186/1471-2466-13-38, PMID: 23758826 PMC3751945

[221] Ram FSF Robinson SM Black PN. Effects of physical training in Asthma: a systematic review. Br J Sports Med. (2000) 34:162–7. doi: 10.1136/bjsm.34.3.162, PMID: 10854014 PMC1763260

[222] Hansen ESH Pitzner-Fabricius A Toennesen LL Rasmusen HK Hostrup M Hellsten Y. Effect of aerobic exercise training on Asthma in adults: a systematic review and Meta-analysis. Eur Respir J. (2020) 56:–2000146. doi: 10.1183/13993003.00146-2020, PMID: 32350100

[223] Arandelović M Stanković I Nikolić M. Swimming and persons with mild Persistant Asthma. ScientificWorldJournal. (2007) 7:1182–8. doi: 10.1100/tsw.2007.221, PMID: 17704850 PMC5900848

[224] Razavi MZ Nazarali P Hanachi P. The effect of an exercise Programme and consumption of vitamin D on performance and respiratory indicators in patients with Asthma. Sport Sci Health. (2011) 2011:5. doi: 10.1007/s11332-011-0102-5

[225] Scott HA Gibson PG Garg ML Pretto JJ Morgan PJ Callister R. Dietary restriction and exercise improve airway inflammation and clinical outcomes in overweight and obese Asthma: a randomized trial. Clin Exp Allergy. (2013) 43:36–49. doi: 10.1111/cea.12004, PMID: 23278879

[226] Shaw I Shaw BS Brown GA. Role of diaphragmatic breathing and aerobic exercise in improving pulmonary function and maximal oxygen consumption in asthmatics. Sci Sports. (2010) 2010:3. doi: 10.1016/j.scispo.2009.10.003

[227] Shaw BS Shaw I. Pulmonary function and abdominal and thoracic kinematic changes following aerobic and inspiratory resistive diaphragmatic breathing training in asthmatics. Lung. (2011) 189:131–9. doi: 10.1007/s00408-011-9281-8, PMID: 21318637

[228] Bacon SL Platts-Mills TAE. Is it time for aerobic exercise to be included in Asthma treatment guidelines? Journal of allergy and clinical immunology. In Pract. (2020) 8:2997–8. doi: 10.1016/j.jaip.2020.08.003, PMID: 33039014 PMC8496979

[229] Turner S Eastwood P Cook A Jenkins S. Improvements in symptoms and quality of life following exercise training in older adults with moderate/severe persistent Asthma. Respiration. (2011) 81:302–10. doi: 10.1159/000315142, PMID: 20501982

[230] Evaristo KB Mendes FAR Saccomani MG Cukier A Carvalho-Pinto RM Rodrigues MR. Effects of aerobic training versus breathing exercises on Asthma control: a randomized trial. J Allergy Clin Immunol Pract. (2020) 8:2989–96. doi: 10.1016/j.jaip.2020.06.042, PMID: 32773365

[231] França-Pinto A Mendes FAR De Carvalho-Pinto RM Agondi RC Cukier A Stelmach R. Aerobic training decreases bronchial Hyperresponsiveness and systemic inflammation in patients with moderate or severe Asthma: a randomised controlled trial. Thorax. (2015) 70:732–9. doi: 10.1136/thoraxjnl-2014-206070, PMID: 26063507

[232] Freitas PD Ferreira PG Analuci S Stelmach R Pinto RC Sage JM. Effects of exercise training in a weight Loss lifestyle intervention on clinical control, quality of life and psychosocial symptoms in obese asthmatics: a RCT. Eur Respir J. (2014) 2014:11.

[233] Dixon AE Holguin F Sood A Salome CM Pratley RE Beuther DA. An official American Thoracic Society workshop report: obesity and Asthma. Proc Am Thorac Soc. (2010) 7:325–35. doi: 10.1513/pats.200903-013ST, PMID: 20844291

[234] Reddel HK Bateman ED Becker A Boulet L-P Cruz AA Drazen JM. A summary of the new GINA strategy: a roadmap to Asthma control. Eur Respir J. (2015) 46:622–39. doi: 10.1183/13993003.00853-2015, PMID: 26206872 PMC4554554

[235] Toennesen LL Meteran H Hostrup M Wium Geiker NR Jensen CB Porsbjerg C. Effects of exercise and diet in nonobese Asthma patients-a randomized controlled trial. J Allergy Clin Immunol Pract. (2018) 6:803–11. doi: 10.1016/j.jaip.2017.09.028, PMID: 29133220

[236] Türk Y Theel W van Huisstede A van de Geijn G-JM Birnie E Hiemstra PS. Short-term and long-term effect of a high-intensity pulmonary rehabilitation Programme in obese patients with Asthma: a randomised controlled trial. Eur Respir J. (2020) 56:1901820. doi: 10.1183/13993003.01820-2019, PMID: 32299852

[237] Mendes FAR Gonçalves RC Nunes MPT Saraiva-Romanholo BM Cukier A Stelmach R. Effects of aerobic training on psychosocial morbidity and symptoms in patients with Asthma: a randomized clinical trial. Chest. (2010) 138:331–7. doi: 10.1378/chest.09-2389, PMID: 20363839

[238] Tsai Y-S Lai F-C Chen S-R Jeng C. The influence of physical activity level on heart rate variability among asthmatic adults. J Clin Nurs. (2011) 20:111–8. doi: 10.1111/j.1365-2702.2010.03397.x, PMID: 21050290

[239] Strine TW Mokdad AH Balluz LS Berry JT Gonzalez O. Impact of depression and anxiety on quality of life, health behaviors, and Asthma control among adults in the United States with Asthma, 2006. J Asthma. (2008) 45:123–33. doi: 10.1080/02770900701840238 18350404

[240] Itkin IH Nacman M. The effect of exercise on the hospitalized asthmatic patient. J Allergy. (1966) 37:253–63. doi: 10.1016/s0021-8707(96)90009-x, PMID: 5219984

[241] Robinson DM Egglestone DM Hill PM Rea HH Richards GN Robinson SM. Effects of a physical conditioning Programme on asthmatic patients. N Z Med J. (1992) 105:253–6. PMID: 1620508

[242] Emtner M Herala M Stålenheim G. High-intensity physical training in adults with Asthma. A 10-week rehabilitation program. Chest. (1996) 109:323–30. doi: 10.1378/chest.109.2.323, PMID: 8620700

[243] Ramos-Campo DJ Andreu-Caravaca L Carrasco-Poyatos M Benito PJ Rubio-Arias JÁ. Effects of circuit resistance training on body composition, strength, and cardiorespiratory fitness in middle-aged and older women: a systematic review and Meta-analysis. J Aging Phys Act. (2021) 30:725–38. doi: 10.1123/japa.2021-0204 34627129

[244] Muñoz-Martínez FA Rubio-Arias JÁ Ramos-Campo DJ Alcaraz PE. Effectiveness of resistance circuit-based training for maximum oxygen uptake and upper-body one-repetition maximum improvements: a systematic review and Meta-analysis. Sports Med. (2017) 47:2553–68. doi: 10.1007/s40279-017-0773-4, PMID: 28822112

[245] Ramos-Campo DJ Andreu Caravaca L Martínez-Rodríguez A Rubio-Arias JÁ. Effects of resistance circuit-based training on body composition, strength and cardiorespiratory fitness: a systematic review and Meta-analysis. Biology (Basel). (2021) 10:377. doi: 10.3390/biology10050377, PMID: 33924785 PMC8145598

[246] Dogra S Jamnik V Baker J. Self-directed exercise improves perceived measures of health in adults with partly controlled Asthma. J Asthma. (2010) 47:972–7. doi: 10.1080/02770903.2010.508857, PMID: 20868317

[247] Bruurs MLJ van der Giessen LJ Moed H. The effectiveness of physiotherapy in patients with Asthma: a systematic review of the literature. Respir Med. (2013) 107:483–94. doi: 10.1016/j.rmed.2012.12.017, PMID: 23333065

[248] Zhang W Wang Q Liu L Yang W Liu H. Effects of physical therapy on lung function in children with Asthma: a systematic review and Meta-analysis. Pediatr Res. (2021) 89:1343–51. doi: 10.1038/s41390-020-0874-x, PMID: 32244247

[249] Eijkemans M Mommers M Draaisma JMT Thijs C Prins MH. Physical activity and Asthma: a systematic review and Meta-analysis. PLoS One. (2012) 7:e50775. doi: 10.1371/journal.pone.0050775, PMID: 23284646 PMC3527462

[250] Bruton A Thomas M. The role of breathing training in Asthma management. Curr Opin Allergy Clin Immunol. (2011) 11:53–7. doi: 10.1097/ACI.0b013e3283423085, PMID: 21150439

[251] Bott J Blumenthal S Buxton M Ellum S Falconer C Garrod R. Guidelines for the physiotherapy Management of the Adult, medical, spontaneously breathing patient. Thorax. (2009) 64:i1–i51. doi: 10.1136/thx.2008.110726, PMID: 19406863

[252] Ram FSF Holloway EA Jones PW. Breathing retraining for Asthma. Respir Med. (2003) 97:501–7. doi: 10.1053/rmed.2002.1472, PMID: 12735667

[253] Olenich S Waterworth G Badger GJ Levy B Israel E Langevin HM. Flexibility and strength training in Asthma: a pilot study. J Asthma. (2018) 55:1376–83. doi: 10.1080/02770903.2017.1414236, PMID: 29420095

[254] Hondras MA Linde K Jones AP. Manual therapy for Asthma. Cochrane Database Syst Rev. (2005) 2:CD001002. doi: 10.1002/14651858.CD001002.pub2, PMID: 15846609 PMC13429289

[255] Silva IS Fregonezi GAF Dias FAL Ribeiro CTD Guerra RO Ferreira GMH. Inspiratory muscle training for Asthma. Cochrane Database Syst Rev. 2013, 2013:CD003792. doi: 10.1002/14651858.CD003792.pub2, PMID: 24014205 PMC7163283

[256] Illi SK Held U Frank I Spengler CM. Effect of respiratory muscle training on exercise performance in healthy individuals: a systematic review and Meta-analysis. Sports Med. (2012) 42:707–24. doi: 10.1007/BF03262290, PMID: 22765281

[257] Chen Y Fu H. Inspiratory muscle training for asthmatic patients: a meta-analysis of randomized controlled studies. Phys Med Rehabil Kurortmedizin. (2022) 2022:1–8.

[258] Duruturk N Acar M Dogrul M. Effect of inspiratory muscle training in the Management of Patients with Asthma. J Cardiopulm Rehabil Prev. (2018) 38:198–203. doi: 10.1097/HCR.0000000000000318, PMID: 29652761

[259] Chung Y Huang TY Liao YH Kuo YC. 12-week inspiratory muscle training improves respiratory muscle strength in adult patients with stable Asthma: a randomized controlled trial. Int J Environ Res Public Health. (2021) 18:3267. doi: 10.3390/ijerph18063267, PMID: 33809922 PMC8004228

[260] Weiner P Magadle R Beckerman M Berar-Yanay N. The relationship among inspiratory muscle strength, the perception of dyspnea and inhaled Beta2-agonist use in patients with Asthma. Can Respir J. (2002) 9:307–12. doi: 10.1155/2002/746808, PMID: 12410322

[261] Alwarith J Kahleova H Crosby L Brooks A Brandon L Levin SM. The role of nutrition in Asthma prevention and treatment. Nutr Rev. (2020) 78:928–38. doi: 10.1093/nutrit/nuaa005, PMID: 32167552 PMC7550896

[262] Kim J-H Ellwood PE Asher MI. Diet and Asthma: looking Back, moving forward. Respir Res. (2009) 10:49. doi: 10.1186/1465-9921-10-49, PMID: 19519921 PMC2703624

[263] Bermudez OI Tucker KL. Trends in dietary patterns of Latin American populations. Cad Saude Publica. (2003) 19:S87–99. doi: 10.1590/s0102-311x2003000700010, PMID: 12886439

[264] Berthon BS Macdonald-Wicks LK Gibson PG Wood LG. Investigation of the association between dietary intake, disease severity and airway inflammation in Asthma. Respirology. (2013) 18:447–54. doi: 10.1111/resp.12015, PMID: 23145908

[265] Jatakanon A Uasuf C Maziak W Lim S Chung KF Barnes PJ. Neutrophilic inflammation in severe persistent Asthma. Am J Respir Crit Care Med. (1999) 160:1532–9. doi: 10.1164/ajrccm.160.5.9806170, PMID: 10556116

[266] Simpson JL Scott R Boyle MJ Gibson PG. Inflammatory subtypes in Asthma: assessment and identification using induced sputum. Respirology. (2006) 11:54–61. doi: 10.1111/j.1440-1843.2006.00784.x, PMID: 16423202

[267] Kim HY Lee HJ Chang Y-J Pichavant M Shore SA Fitzgerald KA. Interleukin-17-producing innate lymphoid cells and the NLRP3 Inflammasome facilitate obesity-associated airway Hyperreactivity. Nat Med. (2014) 20:54–61. doi: 10.1038/nm.3423, PMID: 24336249 PMC3912313

[268] Yusoff NA Hampton SM Dickerson JW Morgan JB. The effects of exclusion of dietary egg and Milk in the Management of Asthmatic Children: a pilot study. J R Soc Promot Heal. (2004) 124:74–80. doi: 10.1177/146642400412400211, PMID: 15067979

[269] Han Y-Y Forno E Brehm JM Acosta-Pérez E Alvarez M Colón-Semidey A. Diet, Interleukin-17, and childhood Asthma in Puerto Ricans. Ann Allergy Asthma Immunol. (2015) 115:288–293.e1. doi: 10.1016/j.anai.2015.07.020, PMID: 26319606 PMC4721241

[270] Woods RK Walters EH Raven JM Wolfe R Ireland PD Thien FCK. Food and nutrient intakes and Asthma risk in Young adults. Am J Clin Nutr. (2003) 78:414–21. doi: 10.1093/ajcn/78.3.414, PMID: 12936923

[271] Haas F Bishop MC Salazar-Schicchi J Axen KV Lieberman D Axen K. Effect of Milk ingestion on pulmonary function in healthy and asthmatic subjects. J Asthma. (1991) 28:349–55. doi: 10.3109/02770909109089462, PMID: 1938769

[272] Woods RK Weiner JM Abramson M Thien F Walters EH. Do dairy products induce bronchoconstriction in adults with Asthma? J Allergy Clin Immunol. (1998) 101:45–50. doi: 10.1016/S0091-6749(98)70192-7, PMID: 9449500

[273] Nguyen MT. Effect of cow Milk on pulmonary function in atopic asthmatic patients. Ann Allergy Asthma Immunol. (1997) 79:62–4. doi: 10.1016/S1081-1206(10)63086-4, PMID: 9236502

[274] Rice JL Romero KM Galvez Davila RM Meza CT Bilderback A Williams DL. Association between adherence to the Mediterranean diet and Asthma in Peruvian children. Lung. (2015) 193:893–9. doi: 10.1007/s00408-015-9792-9, PMID: 26335393 PMC4651981

[275] Guglani L Joseph CL. Asthma and diet: Could food be thy medicine? Indian Pediatr. (2015) 52:21–2. PMID: 25638178 PMC4864955

[276] Lv N Xiao L Ma J. Dietary pattern and Asthma: a systematic review and Meta-analysis. J Asthma Allergy. (2014) 7:105–21. doi: 10.2147/JAA.S49960, PMID: 25143747 PMC4137988

[277] Garcia-Marcos L Castro-Rodriguez JA Weinmayr G Panagiotakos DB Priftis KN Nagel G. Influence of Mediterranean diet on Asthma in children: a systematic review and Meta-analysis. Pediatr Allergy Immunol. (2013) 24:330–8. doi: 10.1111/pai.12071, PMID: 23578354

[278] Romieu I Barraza-Villarreal A Escamilla-Núñez C Texcalac-Sangrador JL Hernandez-Cadena L Díaz-Sánchez D. Dietary intake, lung function and airway inflammation in Mexico City school children exposed to air pollutants. Respir Res. (2009) 10:122. doi: 10.1186/1465-9921-10-122, PMID: 20003306 PMC2806363

[279] Calatayud-Sáez FM Calatayud Moscoso Del Prado B Gallego Fernández-Pacheco JG González-Martín C Alguacil Merino LF. Mediterranean diet and childhood Asthma. Allergol Immunopathol (Madr). (2016) 44:99–105. doi: 10.1016/j.aller.2015.04.007, PMID: 26278484

[280] Lindahl O Lindwall L Spångberg A Stenram A Ockerman PA. Vegan regimen with reduced medication in the treatment of bronchial Asthma. J Asthma. (1985) 22:45–55. doi: 10.3109/02770908509079883, PMID: 4019393

[281] Seyedrezazadeh E Moghaddam MP Ansarin K Vafa MR Sharma S Kolahdooz F. Fruit and vegetable intake and risk of wheezing and Asthma: a systematic review and Meta-analysis. Nutr Rev. (2014) 72:411–28. doi: 10.1111/nure.12121, PMID: 24947126

[282] Willers SM Wijga AH Brunekreef B Scholtens S Postma DS Kerkhof M. Childhood diet and Asthma and atopy at 8 years of age: the PIAMA birth cohort study. Eur Respir J. (2011) 37:1060–7. doi: 10.1183/09031936.00106109, PMID: 21109553

[283] Ellwood P Asher MI García-Marcos L Williams H Keil U Robertson C. Do fast foods cause Asthma, Rhinoconjunctivitis and eczema? Global findings from the international study of Asthma and allergies in childhood (ISAAC) phase three. Thorax. (2013) 68:351–60. doi: 10.1136/thoraxjnl-2012-202285, PMID: 23319429

[284] Wood LG Garg ML Smart JM Scott HA Barker D Gibson PG. Manipulating antioxidant intake in Asthma: a randomized controlled trial. Am J Clin Nutr. (2012) 96:534–43. doi: 10.3945/ajcn.111.032623, PMID: 22854412

[285] Iikura M Yi S Ichimura Y Hori A Izumi S Sugiyama H. Effect of lifestyle on Asthma control in Japanese patients: importance of periodical exercise and raw vegetable diet. PLoS One. (2013) 8:e68290. doi: 10.1371/journal.pone.0068290, PMID: 23874577 PMC3706625

[286] Patel BD Welch AA Bingham SA Luben RN Day NE Khaw K-T. Dietary antioxidants and Asthma in adults. Thorax. (2006) 61:388–93. doi: 10.1136/thx.2004.024935, PMID: 16467075 PMC2111195

[287] Baines KJ Wood LG Gibson PG. The nutrigenomics of Asthma: molecular mechanisms of airway neutrophilia following dietary antioxidant withdrawal. OMICS. (2009) 13:355–65. doi: 10.1089/omi.2009.0042, PMID: 19715394

[288] Koloverou E Panagiotakos DB Pitsavos C Chrysohoou C Georgousopoulou EN Grekas A. Adherence to Mediterranean diet and 10-year incidence (2002-2012) of diabetes: correlations with inflammatory and oxidative stress biomarkers in the ATTICA cohort study. Diabetes Metab Res Rev. (2016) 32:73–81. doi: 10.1002/dmrr.2672, PMID: 26104243

[289] Widmer RJ Flammer AJ Lerman LO Lerman A. The Mediterranean diet, its components, and cardiovascular disease. Am J Med. (2015) 128:229–38. doi: 10.1016/j.amjmed.2014.10.014, PMID: 25447615 PMC4339461

[290] Knekt P Kumpulainen J Järvinen R Rissanen H Heliövaara M Reunanen A. Flavonoid intake and risk of chronic diseases. Am J Clin Nutr. (2002) 76:560–8. doi: 10.1093/ajcn/76.3.560, PMID: 12198000

[291] Hu G Cassano PA. Antioxidant nutrients and pulmonary function: the third National Health and nutrition examination survey (NHANES III). Am J Epidemiol. (2000) 151:975–81. doi: 10.1093/oxfordjournals.aje.a010141, PMID: 10853636

[292] Schünemann HJ Grant BJ Freudenheim JL Muti P Browne RW Drake JA. The relation of serum levels of antioxidant vitamins C and E, retinol and carotenoids with pulmonary function in the general population. Am J Respir Crit Care Med. (2001) 163:1246–55. doi: 10.1164/ajrccm.163.5.2007135, PMID: 11316666

[293] Sies H. Oxidative stress: oxidants and antioxidants. Exp Physiol. (1997) 82:291–5. doi: 10.1113/expphysiol.1997.sp004024, PMID: 9129943

[294] Jin L Yan S Shi B Bao H Gong J Guo X. Effects of vitamin a on the Milk performance, antioxidant functions and immune functions of dairy cows. Anim Feed Sci Technol. (2014) 192:15–23.

[295] Allen S Britton JR Leonardi-Bee JA. Association between antioxidant vitamins and Asthma outcome measures: systematic review and Meta-analysis. Thorax. (2009) 64:610–9. doi: 10.1136/thx.2008.101469, PMID: 19406861

[296] Böhm V Lietz G Olmedilla-Alonso B Phelan D Reboul E Bánati D. From carotenoid intake to carotenoid blood and tissue concentrations – implications for dietary intake recommendations. Nutr Rev. (2021) 79:544–73. doi: 10.1093/nutrit/nuaa008, PMID: 32766681 PMC8025354

[297] Ungurianu A Zanfirescu A Nițulescu G Margină D. Vitamin E beyond its antioxidant label. Antioxidants. (2021) 10:634. doi: 10.3390/antiox10050634, PMID: 33919211 PMC8143145

[298] Cook-Mills JM Abdala-Valencia H Hartert T. Two faces of vitamin E in the lung. Am J Respir Crit Care Med. (2013) 188:279–84. doi: 10.1164/rccm.201303-0503ED, PMID: 23905522 PMC3778733

[299] Chen X Kang Y-B Wang L-Q Li Y Luo Y-W Zhu Z. Addition to inhaled corticosteroids of leukotriene receptor antagonists versus theophylline for symptomatic Asthma: a Meta-analysis. J Thorac Dis. (2015) 7:644–52. doi: 10.3978/j.issn.2072-1439.2015.04.12, PMID: 25973230 PMC4419319

[300] Carr AC Maggini S. Vitamin C and immune function. Nutrients. (2017) 9:11. doi: 10.3390/nu9111211, PMID: 29099763 PMC5707683

[301] Hemilä H. The effect of vitamin C on bronchoconstriction and respiratory symptoms caused by exercise: a review and statistical analysis. Allergy Asthma Clin Immunol Clin Immunol. (2014) 10:58. doi: 10.1186/1710-1492-10-58, PMID: 25788952 PMC4363347

[302] Hemilä H. Vitamin C may alleviate exercise-induced bronchoconstriction: a meta-analysis. BMJ Open. (2013) 3:e002416. doi: 10.1136/bmjopen-2012-002416, PMID: 23794586 PMC3686214

[303] Olin JT Wechsler ME. Asthma: pathogenesis and novel drugs for treatment. BMJ. (2014) 349:g5517. doi: 10.1136/bmj.g5517, PMID: 25420994

[304] Hemilä H. Vitamin C and common cold-induced Asthma: a systematic review and statistical analysis. Allergy, asthma, Clin Immunol. (2013) 9:46. doi: 10.1186/1710-1492-9-46, PMID: 24279478 PMC4018579

[305] Tecklenburg SL Mickleborough TD Fly AD Bai Y Stager JM. Ascorbic acid supplementation attenuates exercise-induced bronchoconstriction in patients with Asthma. Respir Med. (2007) 101:1770–8. doi: 10.1016/j.rmed.2007.02.014, PMID: 17412579

[306] Tapas AR Sakarkar DM Kakde RB. Flavonoids as nutraceuticals: a review. Trop J Pharm Res. (2008) 7:1089–99. doi: 10.4314/tjpr.v7i3.14693

[307] Tabak C Arts IC Smit HA Heederik D Kromhout D. Chronic obstructive pulmonary disease and intake of Catechins, Flavonols, and flavones: the MORGEN study. Am J Respir Crit Care Med. (2001) 164:61–4. doi: 10.1164/ajrccm.164.1.2010025, PMID: 11435239

[308] Gong J-H Shin D Han S-Y Park S-H Kang M-K Kim J-L. Blockade of airway inflammation by Kaempferol via disturbing Tyk-STAT signaling in airway epithelial cells and in asthmatic mice. Evid Based Complement Alternat Med. (2013) 2013:250725. doi: 10.1155/2013/250725, PMID: 23737822 PMC3662111

[309] Cho I-H Gong J-H Kang M-K Lee E-J Park JHY Park S-J. Astragalin inhibits airway Eotaxin-1 induction and epithelial apoptosis through modulating oxidative stress-responsive MAPK signaling. BMC Pulm Med. (2014) 14:122. doi: 10.1186/1471-2466-14-122, PMID: 25069610 PMC4118077

[310] Jeong J-H An JY Kwon YT Rhee JG Lee YJ. Effects of low dose quercetin: cancer cell-specific inhibition of cell cycle progression. J Cell Biochem. (2009) 106:73–82. doi: 10.1002/jcb.21977, PMID: 19009557 PMC2736626

[311] Verma R Kushwah L Gohel D Patel M Marvania T Balakrishnan S. Evaluating the ameliorative potential of quercetin against the bleomycin-induced pulmonary fibrosis in Wistar rats. Pulm Med. (2013) 2013:921724. doi: 10.1155/2013/921724, PMID: 24396596 PMC3875129

[312] Liu H Xue J-X Li X Ao R Lu Y. Quercetin liposomes protect against radiation-induced pulmonary injury in a murine model. Oncol Lett. (2013) 6:453–9. doi: 10.3892/ol.2013.1365, PMID: 24137346 PMC3789113

[313] Huang R Zhong T Wu H. Quercetin protects against lipopolysaccharide-induced acute lung injury in rats through suppression of inflammation and oxidative stress. Arch Med Sci. (2015) 11:427–32. doi: 10.5114/aoms.2015.50975, PMID: 25995762 PMC4424260

[314] Stone J Hinks LJ Beasley R Holgate ST Clayton BA. Reduced selenium status of patients with Asthma. Clin Sci (Lond). (1989) 77:495–500. doi: 10.1042/cs0770495, PMID: 2582721

[315] Heffner JE Repine JE. Pulmonary strategies of antioxidant defense. Am Rev Respir Dis. (1989) 140:531–54. doi: 10.1164/ajrccm/140.2.531, PMID: 2669581

[316] Burr ML Butland BK King S Vaughan-Williams E. Changes in Asthma prevalence: two surveys 15 years apart. Arch Dis Child. (1989) 64:1452–6. doi: 10.1136/adc.64.10.1452, PMID: 2817930 PMC1792778

[317] Burney PG Chinn S Rona RJ. Has the prevalence of Asthma increased in children? Evidence from the National Study of health and growth 1973-86. BMJ. (1990) 300:1306–10. doi: 10.1136/bmj.300.6735.1306, PMID: 2369661 PMC1663026

[318] Romieu I Trenga C. Diet and obstructive lung diseases. Epidemiol Rev. (2001) 23:268–87. doi: 10.1093/oxfordjournals.epirev.a000806, PMID: 12192737

[319] Black PN Sharpe S. Dietary fat and Asthma: is there a connection? Eur Respir J. (1997) 10:6–12. doi: 10.1183/09031936.97.10010006, PMID: 9032484

[320] Platts-Mills TA. The role of immunoglobulin E in allergy and Asthma. Am J Respir Crit Care Med. (2001) 164:S1–5. doi: 10.1164/ajrccm.164.supplement_1.2103024, PMID: 11704610

[321] Stoodley I Garg M Scott H Macdonald-Wicks L Berthon B Wood L. Higher Omega-3 index is associated with better Asthma control and lower medication dose: a cross-sectional study. Nutrients. (2019) 12:74. doi: 10.3390/nu12010074, PMID: 31892115 PMC7019867

[322] Bozzetto S Carraro S Giordano G Boner A Baraldi E. Asthma, allergy and respiratory infections: the vitamin D hypothesis. Allergy. (2012) 67:10–7. doi: 10.1111/j.1398-9995.2011.02711.x, PMID: 21933195

[323] Adams JS Hewison M. Unexpected actions of vitamin D: new perspectives on the regulation of innate and adaptive immunity. Nat Clin Pract Endocrinol Metab. (2008) 4:80–90. doi: 10.1038/ncpendmet0716, PMID: 18212810 PMC2678245

[324] Ly NP Litonjua A Gold DR Celedón JC. Gut microbiota, probiotics, and vitamin D: interrelated exposures influencing allergy, Asthma, and obesity? J Allergy Clin Immunol. (2011) 127:1086–7. doi: 10.1016/j.jaci.2011.02.015, PMID: 21419479 PMC3085575

[325] Lobo V Patil A Phatak A Chandra N. Free radicals, antioxidants and functional foods: impact on human health. Pharmacogn Rev. (2010) 4:118–26. doi: 10.4103/0973-7847.70902, PMID: 22228951 PMC3249911

[326] Bener A Ehlayel MS Tulic MK Hamid Q. Vitamin D deficiency as a strong predictor of Asthma in children. Int Arch Allergy Immunol. (2012) 157:168–75. doi: 10.1159/000323941, PMID: 21986034

[327] Checkley W Robinson CL Baumann LM Hansel NN Romero KM Pollard SL. 25-Hydroxy vitamin D levels are associated with childhood Asthma in a population-based study in Peru. Clin Exp Allergy. (2015) 45:273–82. doi: 10.1111/cea.12311, PMID: 24666565 PMC4176605

[328] Brehm JM Schuemann B Fuhlbrigge AL Hollis BW Strunk RC Zeiger RS. Serum vitamin D levels and severe Asthma exacerbations in the childhood Asthma management program study. J Allergy Clin Immunol. (2010) 126:52–8.e5. doi: 10.1016/j.jaci.2010.03.043, PMID: 20538327 PMC2902692

[329] Brehm JM Celedón JC Soto-Quiros ME Avila L Hunninghake GM Forno E. Serum vitamin D levels and markers of severity of childhood Asthma in Costa Rica. Am J Respir Crit Care Med. (2009) 179:765–71. doi: 10.1164/rccm.200808-1361OC, PMID: 19179486 PMC2675563

[330] Yorke J Fleming SL Shuldham CM. Psychological interventions for adults with Asthma. Cochrane Database Syst Rev. (2006) 1:CD002982. doi: 10.1002/14651858.CD002982.pub3, PMID: 16437449 PMC7004249

[331] Lehrer P Feldman J Giardino N Song H-S Schmaling K. Psychological aspects of Asthma. J Consult Clin Psychol. (2002) 70:691–711. doi: 10.1037//0022-006x.70.3.691 12090377

[332] Bender B Milgrom H Rand C Ackerson L. Psychological factors associated with medication nonadherence in asthmatic children. J Asthma. (1998) 35:347–53. doi: 10.3109/02770909809075667, PMID: 9669828

[333] Cooley C Park Y Ajilore O Leow A Nyenhuis SM. Impact of interventions targeting anxiety and depression in adults with Asthma. J Asthma. (2022) 59:273–87. doi: 10.1080/02770903.2020.1847927, PMID: 33176512 PMC8221364

[334] Pourdowlat G Hejrati R Lookzadeh S. The effectiveness of relaxation training in the quality of life and anxiety of patients with Asthma. Adv Respir Med. (2019) 87:146–51. doi: 10.5603/ARM.2019.0024, PMID: 31282555

[335] Thomas M Bruton A Moffat M Cleland J. Asthma and psychological dysfunction. Prim Care Respir J. (2011) 20:250–6. doi: 10.4104/pcrj.2011.00058, PMID: 21674122 PMC6549858

[336] Roberts G Vazquez-Ortiz M Knibb R Khaleva E Alviani C Angier E. EAACI guidelines on the effective transition of adolescents and Young adults with allergy and Asthma. Allergy. (2020) 75:2734–52. doi: 10.1111/all.14459, PMID: 32558994

[337] Ramsey RR Plevinsky JM Kollin SR Gibler RC Guilbert TW Hommel KA. Systematic review of digital interventions for pediatric Asthma management. J Allergy Clin Immunol Pract. (2020) 8:1284–93. doi: 10.1016/j.jaip.2019.12.013, PMID: 31870809 PMC7152564

[338] Stubbs MA Clark VL McDonald VM. Living well with severe Asthma. Breathe (Sheff). (2019) 15:e40–9. doi: 10.1183/20734735.0165-2019, PMID: 31777564 PMC6876145

[339] Freeman AT Hill D Newell C Moyses H Azim A Knight D. Patient perceived barriers to exercise and their clinical associations in difficult Asthma. Asthma Res Pract. (2020) 6:5. doi: 10.1186/s40733-020-00058-6, PMID: 32537235 PMC7285728

[340] Paoletti G Keber E Heffler E Malipiero G Baiardini I Canonica GW. Effect of an educational intervention delivered by pharmacists on adherence to treatment, disease control and lung function in patients with Asthma. Respir Med. (2020) 174:106199. doi: 10.1016/j.rmed.2020.106199, PMID: 33120195

[341] Schuers M Chapron A Guihard H Bouchez T Darmon D. Impact of non-drug therapies on Asthma control: a systematic review of the literature. Eur J Gen Pract. (2019) 25:65–76. doi: 10.1080/13814788.2019.1574742, PMID: 30849253 PMC6493294

[342] Barnthouse M Jones BL. The impact of environmental chronic and toxic stress on Asthma. Clin Rev Allergy Immunol. (2019) 57:427–38. doi: 10.1007/s12016-019-08736-x, PMID: 31079340

[343] Boggiss AL Consedine NS Brenton-Peters JM Hofman PL Serlachius AS. A systematic review of gratitude interventions: effects on physical health and health behaviors. J Psychosom Res. (2020) 135:110165. doi: 10.1016/j.jpsychores.2020.110165, PMID: 32590219

[344] Lycett H Wildman E Raebel EM Sherlock J-P Kenny T Chan AHY. Treatment perceptions in patients with Asthma: synthesis of factors influencing adherence. Respir Med. (2018) 141:180–9. doi: 10.1016/j.rmed.2018.06.032, PMID: 30053965

[345] Murray B O’Neill M. Supporting self-Management of Asthma through patient education. Br J Nurs. (2018) 27:396–401. doi: 10.12968/bjon.2018.27.7.396, PMID: 29634337

[346] Knibb RC Alviani C Garriga-Baraut T Mortz CG Vazquez-Ortiz M Angier E. The effectiveness of interventions to improve self-Management for Adolescents and Young Adults with allergic conditions: a systematic review. Allergy. (2020) 75:1881–98. doi: 10.1111/all.14269, PMID: 32159856

[347] Ramsey RR Caromody JK Voorhees SE Warning A Cushing CC Guilbert TW. A systematic evaluation of Asthma management apps examining behavior change techniques. J Allergy Clin Immunol Pract. (2019) 7:2583–91. doi: 10.1016/j.jaip.2019.03.041, PMID: 30954644 PMC6776707

[348] Zarneshan A. The efficacy of aerobic and breathing exercise training on Asthma control and physical-psychological health promotion in women with Asthma. Iran J Health Educ Health Promot. (2018) 6:179–88.

[349] Pateraki E Vance Y Morris PG. The interaction between Asthma and anxiety: An interpretative phenomenological analysis of Young People’s experiences. J Clin Psychol Med Settings. (2018) 25:20–31. doi: 10.1007/s10880-017-9528-5, PMID: 29322289

[350] Schakel L Veldhuijzen DS Crompvoets PI Bosch JA Cohen S van Middendorp H. Effectiveness of stress-reducing interventions on the response to challenges to the immune system: a Meta-analytic review. Psychother Psychosom. (2019) 88:274–86. doi: 10.1159/000501645, PMID: 31387109 PMC6878733

[351] Dougru H Sürer-Adanir A Özatalay E. Psychopathology, health-related quality-of-life and parental attitudes in pediatric Asthma. J Asthma. (2019) 56:1204–11. doi: 10.1080/02770903.2018.1531995, PMID: 30335531

[352] Kosse RC Bouvy ML Belitser SV De Vries TW Van Der Wal PS Koster ES. Effective engagement of adolescent Asthma patients with Mobile health--supporting medication adherence. JMIR Mhealth Uhealth. (2019) 7:e12411. doi: 10.2196/12411, PMID: 30916664 PMC6456831

[353] Salim H Ramdzan SN Ghazali SS Lee PY Young I McClatchey K. A systematic review of interventions addressing limited health literacy to improve Asthma self-management. J Glob Health. (2020) 10:10427. doi: 10.7189/jogh.10.010428, PMID: 32566166 PMC7298737

[354] Anastasia P Eleni T Eleftheria M Xenia N Eygenia P Kyriakos S. Depression levels influence the rate of Asthma exacerbations in females. J Pers Med. (2021) 11:586. doi: 10.3390/jpm11060586, PMID: 34205619 PMC8235599

[355] Ong ASE Chan AKW Sultana R Koh MS. Impact of psychological impairment on quality of life and work impairment in severe Asthma. J Asthma. (2021) 58:1544–53. doi: 10.1080/02770903.2020.1808989, PMID: 32777181

[356] Haghighati MA Vali L Goudarzi R Samareh Fekri M Ghorbani Nia R. Perceived Asthma control care and health care participation in patients with Asthma. Tanaffos. (2021) 20:109–15. PMID: 34976081 PMC8710219

[357] Sheha DS Abdel-Rehim AS Abdel-Latif OM Abdelkader MA Raafat RH Sallam SA. Level of Asthma control and mental health of Asthma patients during lockdown for COVID-19: a cross-sectional survey. Egypt J Bronchol. (2021) 15:1–10.

[358] Luyster FS Strollo PJJ Holguin F Castro M Dunican EM Fahy J. Association between insomnia and Asthma burden in the severe Asthma research program (SARP) III. Chest. (2016) 150:1242–50. doi: 10.1016/j.chest.2016.09.020, PMID: 27720882 PMC5310183

[359] Amelink M Hashimoto S Spinhoven P Pasma HR Sterk PJ Bel EH. Anxiety, depression and personality traits in severe, prednisone-dependent asthma. Respir Med. (2014) 108:438–44. doi: 10.1016/j.rmed.2013.12.012, PMID: 24462260

[360] Di Marco F Verga M Santus P Giovannelli F Busatto P Neri M. Close correlation between anxiety, depression, and Asthma control. Respir Med. (2010) 104:22–8. doi: 10.1016/j.rmed.2009.08.005, PMID: 19733042

